# The effect of a community-based health behaviour intervention on health-related quality of life in people with Type 2 diabetes in Nepal: a Cluster Randomized Controlled Trial

**DOI:** 10.1007/s11136-025-03971-6

**Published:** 2025-04-07

**Authors:** Ashmita Karki, Corneel Vandelanotte, M. Mamun Huda, Lal B. Rawal

**Affiliations:** 1 School of Health, Medical and Applied Sciences, Appleton Institute, Central Queensland University, Building 7, Bruce Highway, Rockhampton, QLD 4702 Australia; 2 Rural Health Research Institute, Charles Sturt University, Orange, NSW Australia; 3 Translational Health Research Institute (THRI), Western Sydney University, Sydney, Australia

**Keywords:** Health-related quality of life, Type 2 diabetes, EuroQOL, EQ5D

## Abstract

**Purpose:**

Little is known about the effectiveness of health behaviour intervention in improving health-related quality of life (HRQOL) despite HRQOL being an important outcome in Type 2 diabetes (T2DM) management. This study examined the effectiveness of a culturally appropriate health behavioural intervention in improving HRQOL of people with T2DM in Nepal.

**Methods:**

A cluster randomized controlled trial was conducted among 481 people with T2DM from 30 randomly selected healthcare facilities in Kavrepalanchok and Nuwakot districts. The intervention group received 12 culturally tailored and group-based intervention sessions for six months whereas, the control group received usual care. The primary outcomes of this study were changes in the EuroQOL (EQ5D-3L) index score and EuroQOL visual analogue scale (EQVAS) score between baseline and six-month post-intervention. The intervention effect was assessed using generalized estimating equation models.

**Results:**

At six-months post-intervention, there was a positive and statistically significant effect on EQVAS (β_1_ = 3.61, 95%CI: 0.05, 7.17) in the intervention group compared to control group. No statistically significant effect was observed in EQ5D-3L index score. A statistically significant increase in EQVAS score of 0.5 was observed per session attended by the study participants (β_1_ = 0.49, 95%CI: 0.01, 0.98).

**Conclusion:**

The health behaviour intervention led to improved HRQOL. An increase in the number of intervention sessions attended was significantly associated with higher EQVAS scores, emphasizing the need for longer-term and engaging interventions that are well adhered to. Longer term assessment of change in HRQOL outcomes are needed when actual changes in HRQOL are more likely to be observed.

**Trial registration:**

Australia and New Zealand Clinical Trial Registry (ACTRN12621000531819).

**Supplementary Information:**

The online version contains supplementary material available at 10.1007/s11136-025-03971-6.

## Plain English summary

Little is known about the effectiveness of health behaviour intervention in improving health-related quality of life (HRQOL) of people with Type 2 diabetes mellitus (T2DM) despite HRQOL being an important outcome in T2DM management. This study examined the effectiveness of a culturally appropriate health behavioural intervention in improving HRQOL of people with T2DM in rural Nepal. Culturally tailored community-based health behaviour intervention comprising twelve intervention modules, targeted for the management of Type 2 diabetes in rural Nepal improved HRQOL of the study participants. Higher attendance to intervention sessions was significantly associated with higher HRQOL, emphasizing the need for designing intervention that are engaging for participants and are well adhered too. More detailed and well-designed studies with longer term intervention are required to understand what changes in the HRQOL values are of clinical importance for people living with T2DM. Further, actual changes in HRQOL are more likely to be observed in the long term rather than immediately, hence, longer term assessment of change in HRQOL outcomes are needed rather than immediate post-intervention assessment.

## Introduction

The burden of Type 2 diabetes mellitus (T2DM) is steadily rising in Nepal, from a prevalence of around 4% in 2000s to the current estimated prevalence of 8.5% [1]. Management of a chronic condition like T2DM is often challenging, more so in a resource-constraint nation like Nepal; however, community-based health behaviour interventions have been proven effective in managing T2DM in under-resourced settings, where T2DM medication and treatment costs could be overwhelming to T2DM patients [2]. These interventions include people-centred behavioural programs such as dietary and/or physical activity programs, self-management education on medication adherence, glucose monitoring, stress management, care of feet, eyes and mouth, etc. Moreover, these interventions need to be culturally adapted and foster local capacity building to ensure the effectiveness, sustainability, community acceptance and ownership of the intervention.

To date, most trials have assessed their effectiveness against quantifiable and objective outcomes such as reduction in glucose levels and lipid profiles, but subjective outcomes such as quality of life have often been ignored. However, over the years, it has become increasingly apparent that metabolic biomarkers alone do not comprehensively capture the health status of people living with T2DM [3]. The effect of the condition and its management on one’s physical, psychological and social aspects of life, otherwise known as health-related quality of life (HRQOL), is an equally important element of overall function, health and well-being [4]. Recognising this much-needed shift from a disease-centred approach to a patient-centred approach in the T2DM management paradigm, recent trials with behavioural interventions have extended their aim from establishing metabolic control to also improving HRQOL [5].

Previous meta-analyses have quantified the effectiveness of diabetes self-management interventions in improving HRQOL among those who received the interventions versus those who did not, yielding a small but significant effect size of 0.28 [6] and 0.26 [7] respectively. These effect sizes mean that the self-management interventions resulted in improved HRQOL outcomes in the T2DM population. Even though the effect sizes were small, which could be attributed to the use of varying kinds of tools to measure a complex phenomenon like HRQOL, these meta-analyses argued that any statistically significant improvement, albeit small, could still translate to meaningful benefits provided that the interventions resulted in metabolic control [6, 7]. A recent systematic review and meta-analysis of 45 RCTs conducted in developing countries identified health behaviour interventions as effective strategies to enhance the HRQOL of people with T2DM, however, the moderate certainty of evidence highlighted the need for more well-designed trials with methodological rigour in order to establish the relation between such behavioural interventions and HRQOL [8]. A cluster randomized controlled trial of 244 people with T2DM in a semi-urban Western Nepal reported that a female community health volunteer (FCHV)-delivered intervention was effective in lowering the mean fasting blood glucose level in the intervention group by 27.90 mg/dL compared to control group in a year [9]. However, the trial did not report the intervention effectiveness in terms of HRQOL. Moreover, the findings were limited due to small sample size and use of only fasting blood glucose without other glycaemic indexes such as glycated haemoglobin (HbA1c) [9]. Another recent RCT conducted in a hospital-based setting in Nepal demonstrated that a dietitian-led self-management intervention which involved nutrition counselling and individualised diet plan significantly reduced scores on the Problems Areas in diabetes (PAID) scale among people with T2DM, meaning the intervention was effective in significantly reducing negative emotions and distress associated with the condition [10]. However, the findings were limited due to small sample size (n = 156) and single centred setting of the study. As such, there is limited evidence whether such diabetes management interventions are effective in improving diabetes-related or HRQOL outcomes in rural population of Nepal.

Although the never-ending need for lifestyle adjustments is known to lower T2DM patients’ HRQOL, behavioural measures that can potentially avert diabetes-related comorbidities and complications have also been known to enhance their HRQOL. In light of this, a culturally adapted community-based lifestyle intervention for diabetes management (Co-LID study) [11] with a cluster randomized design, was conducted in rural Nepal to examine the effectiveness of culturally-appropriate health behavioural interventions in improving T2DM outcomes—primarily, HbA1c level; and secondarily, HRQOL, mental health outcomes, self-care behaviours, and health care utilization. The aim of this study was to examine the effect of a community-based health behaviour intervention on HRQOL of people with T2DM in Nepal.

## Methods

### Design and setting

A prospective two-arm cluster randomized controlled trial (RCT) was conducted for a period of six months in two districts of Bagmati province of Nepal—Kavrepalanchowk and Nuwakot. A cluster randomized design was used to minimise contamination and the possibility of selection bias that could have occurred due to preference of the participants or health service providers. The study setting for this study comprised of 30 community health centres in rural and peri-urban areas of these districts. A detailed study protocol has been published elsewhere [11]. The study follows the Consolidated Standards of Reporting Trials (CONSORT) 2010 statement: extension to cluster randomised trials for reporting of its findings [12].

### Participants and eligibility

The Co-LID study was conducted among people aged 30–70 years with clinically diagnosed T2DM i.e., HbA1c > 6.5% or fasting plasma glucose (FPG) > 7.0 mmol/l based on the American Diabetes Association and WHO criteria for diagnosis and classification of diabetes [13, 14]. Those below the age of 30 years, clinically diagnosed with Type 1 diabetes mellitus, currently pregnant, or with cognitive impairment who were unable to respond the questions were excluded from the study.

### Sampling

A stratified random sample of 20 healthcare centres from Kavrepalanchowk and 10 from Nuwakot districts were selected. The unit of sampling or clusters for this study were health centres from the selected districts. A detailed sampling process is reported in the study protocol [11]. Out of 4748 people screened from 30 clusters in Nuwakot and Kavrepalanchowk, 619 had a clinical diagnosis of T2DM and were assessed for eligibility. Of them, 481 consented to participate in the study and were enrolled and randomised.

### Sample size calculation

The calculated sample size in the overarching intervention was 414, obtained from 30 clusters in Kavrepalanchowk and Nuwakot districts, selected based on access and feasibility: 15 clusters randomly allocated in the intervention arm and 15 in the usual care (control) arm with an average of 12 participants in each cluster. The sample size in the main study was calculated with the aim to reduce the mean HbA1c levels from 7.5% at baseline to 7.0% at 6 months follow-up among the intervention participants compared with estimated no or minimal changes in participants of usual care arm (HbA1c levels 7.5%), with at least 80% power and at 95% confidence interval assuming an intra-class correlation coefficient (ICC) of 0.050 as noted in a previous study conducted in an Asian setting [15] and an estimated HbA1c mean standard deviation (SD) of 1.3 as reported in previous studies that used diabetes self-management programs [16]. With the adjustment of possible 15% attrition rate from baseline to 6-month follow up, a total of 414 samples were calculated. Hence, the minimum sample size required for this study was 414. All trial participants (n = 481) were included in this study.

### Randomization

The health centre (as a cluster) was considered as a unit of sample for randomization. Thirty health centres were randomly allocated as intervention or usual care clustered groups with the sample allocation ratio of 1:1 using simple randomization technique by a statistician not involved in the study. Due to the nature of the study, neither participants nor those who delivered the interventions were blinded. However, the statistician was blinded to group assignment.

### Intervention

The primary aim of the lifestyle intervention was to achieve reduction in HbA1c level among people with T2DM in the intervention arm at 6-month follow-up compared to no possible change or increment of HbA1c level in people with T2DM in the usual care arm. To ensure participants’ homogeneity, all participants received a two-hour education session on diabetes self-management prior to randomization. After randomization, the intervention group received a culturally tailored and locally based group intervention program consisting of fortnightly sessions that covered 12 modules over six months, accompanied by ongoing support. These modules, each lasting for 60 min, included information on diabetes literacy, diabetes medications, physical activity, dietary modification, management of stress and depression, smoking cessation and reducing harmful alcohol consumption, monitoring blood sugar level, foot care, oral health, quality of life, health care utilization, and social and emotional support (Supplementary File S1). Further, group-based sessions on local practices such as cooking, group exercise, yoga, were conducted to complement the primary intervention modules and enhance participants’ locally acquired skills related to meal preparation and group exercises. Throughout the delivery of the intervention, culturally adapted practices were adopted. This included culturally tailored dietary education respecting traditional eating habits and local recipes; meal preparation using local produces; behavioural education on traditional physical activities such as farming and household chores; culturally relevant approaches such as use of local language, narratives, metaphors, storytelling and knowledge sharing; provision of pictorial book on diabetes management and prevention of complication in Nepali language; empathetic listening and counselling; and family and social support. Furthermore, the intervention program was delivered by trained community health workers (CHWs) and peer supporters, who later facilitated the group-based intervention to the intervention group participants. CHWs are the frontline healthcare workers in government health facilities in Nepal [17] and peer supporters are participants from the intervention group who are committed to managing their own health behaviours while also assisting others. CHWs are the primary point of contact for health-related issues among the local population; therefore, the Training of Trainers model was adopted for local capacity building of CHWs in the two districts (Health Assistant, Auxiliary Nurse Mid-wife or Auxiliary Health Worker) in delivering the intervention sessions. This also helped foster the cultural appropriateness of the intervention. A comprehensive training guide for CHWs and peer supporters is provided in the appendix (Supplementary File S2). Additionally, to ensure regular contact between CHWs and participants and maintenance of self-care behaviours learnt in the sessions, CHWs conducted fortnightly telephone calls for the first three months and monthly calls for the rest of the months, along with online pictorial and audio-visual messages on lifestyle intervention in Nepali language sent to the participants’ phones. The intervention group also received locally available care, which was standard diabetes management and care available at the respective health centres, in accordance with the standard practice and guidelines.

A description of the intervention modules is attached in the appendix (Supplementary File S1) and also published in the study protocol [11]. On the other hand, the participants in the control group received the usual care with no lifestyle intervention by CHWs and the peer supporters. However, they received a pictorial brochure on diabetes education in Nepali language so that they were not deprived of awareness. Follow-up was conducted for the participants of both intervention and usual care groups at six months.

### Measures

A structured questionnaire was prepared to collect socio-demographic and diabetes-related information from study participants. Health-related quality of life was measured using a Nepali-translated version of EuroQol 5D (EQ5D) tool, which consists of 2 parts—the EQ5D-3L descriptive system and the EuroQol Visual Analogue Scale (EQVAS) [18]. The tool showed a good reliability in this study with a Cronbach’s alpha of 0.75. The descriptive system has 5 dimensions (mobility, self‑care, usual activities, pain/discomfort, and anxiety/depression) and each dimension has 3 response levels (no problems, moderate problems, and extreme problems). The participants’ level of reported problems on each dimension were combined to create a 5-digit number for each participant, known as their health state. EQ5D-3L generates 243 (3^5^) various health states, with 11,111 being the best health state (full health) and 33,333 being the worst health state. If a country has a reference value set, then the health states can be converted to a single utility value (the EQ5D index score). Since there is no EQ5D reference value set for Nepal, the 3L utilities were generated using the reverse crosswalk index value calculator [19] with 5L value sets for a neighbouring country, India, for the purpose of this study. Similarly, an EQVAS tool, a 20 cm wide 100-point scale, was used to allow respondents to rate their health on that day from the worst imaginable health state (0) to the best imaginable health state (100). Measurements of covariates along with the tools used and their psychometric properties are presented in the appendix (Supplementary Table S3). The survey questionnaire was translated into Nepali language to best fit in Nepalese context and pre-tested prior to baseline data collection. The questionnaire was then back translated to English by two independent translators.

### Outcome measures

The primary outcomes of this study were changes in the EQ5D-3L score and EQVAS score in the intervention group compared to usual care group at the end of six-month intervention against the baseline status. The outcomes were measured at the individual participant level.

### Statistical analysis

The baseline data were analyzed using descriptive summaries such as frequencies and percentages for categorical variables, means and standard deviations for normally distributed continuous variables, and medians and interquartile ranges (IQR) for non-normally distributed continuous variable. The difference between the intervention and usual care groups in terms of socio-demographic and diabetes-related variables were measured at baseline using standardized mean differences (SMDs). An SMD of less than 0.1 was considered to indicate negligible difference [20].

The outcome variables of this study were EQ5D-3L score and EQVAS score. We assessed the outcome of this study using Intention-to-Treat (ITT) analysis in accordance with the study protocol. We handled missing data due to loss of follow-up using a multiple imputation (MI) technique (see below).

First, we computed the proportion of outcomes across various groups, including baseline, follow-up, intervention, and control; and compared the proportions across the groups using a t-test. Then, we employed a difference-in-difference (DID) regression model to assess the effect of the intervention on the study outcomes. This model considered baseline differences in outcomes and variations between the intervention and control groups in terms of the distribution of covariates.

We used a generalized estimating equation (GEE) model to account for within-cluster correlation. First, we fitted the unadjusted model by considering only the time and group variables in the model and got the unadjusted intervention effect. The model was then extended further by adjusting for variables that differed significantly (p-value < 0.200) at baseline between intervention and control group to get the adjusted intervention effect. The effect estimates were reported as coefficients. A p-value < 0.05 was considered statistically significant.

Furthermore, to explore the association between the number of intervention sessions attended and HRQOL among participants who received interventions, a subset of the intervention group was analysed. Both adjusted and unadjusted GEE models with a binary logit function were constructed for this association analysis, where EQ5D-3L and EQVAS served as outcome variables and the number of sessions attended was the primary exposure of interest.

The statistical analyses originally proposed in the trial registration included a random effects regression model and analysis of covariance (ANCOVA). However, we opted for a DID GEE regression model in the final analysis because a) our primary interest was population-averaged effects, which is estimated by GEE, rather than cluster-specific effects, estimated by random effects regression model; b) GEE more effectively accounts for within-subject correlations than ANCOVA, given the longitudinal nature of our data; c) DID controls for baseline differences between groups accounting for differential trends thereby enhancing causal inference; d) GEE estimates robust standard errors ensuring valid inferences even when the true correlation structure is mis-specified versus random effects model, which assumes that random effects (e.g., subject- or cluster-specific variations) follow a normal distribution; and e) GEE addresses missing data providing more robust estimates under a missing at random assumption compared to the random effects model.

All statistical analyses were performed using Stata, version 18.

#### Multiple imputation in ITT analysis

We employed MI to estimate a set of plausible values for missing data resulting from loss of follow-up and created a "complete" dataset using the distribution of observed data. To address the different variable types (e.g., binary, ordinal, continuous), we used chained equations, a sequence of univariate imputation methods with fully conditional specification (FCS) of prediction equations using the STATA command—mi impute chained. The imputation routine consisted of 1000 iterations to create 20 imputed data sets. The imputation validity was assessed by comparing distributions of covariates before and after imputation and imputation accuracy was assessed using the following parameters: Relative Increase in Variance, Fraction of Missing Information, Degrees of Freedom, Relative Efficiency, as well as the between-imputation and the within-imputation variance estimates. All the effects of intervention analysis were performed on missing imputed samples to get the ITT estimates.

## Results

**Fig. 1:**
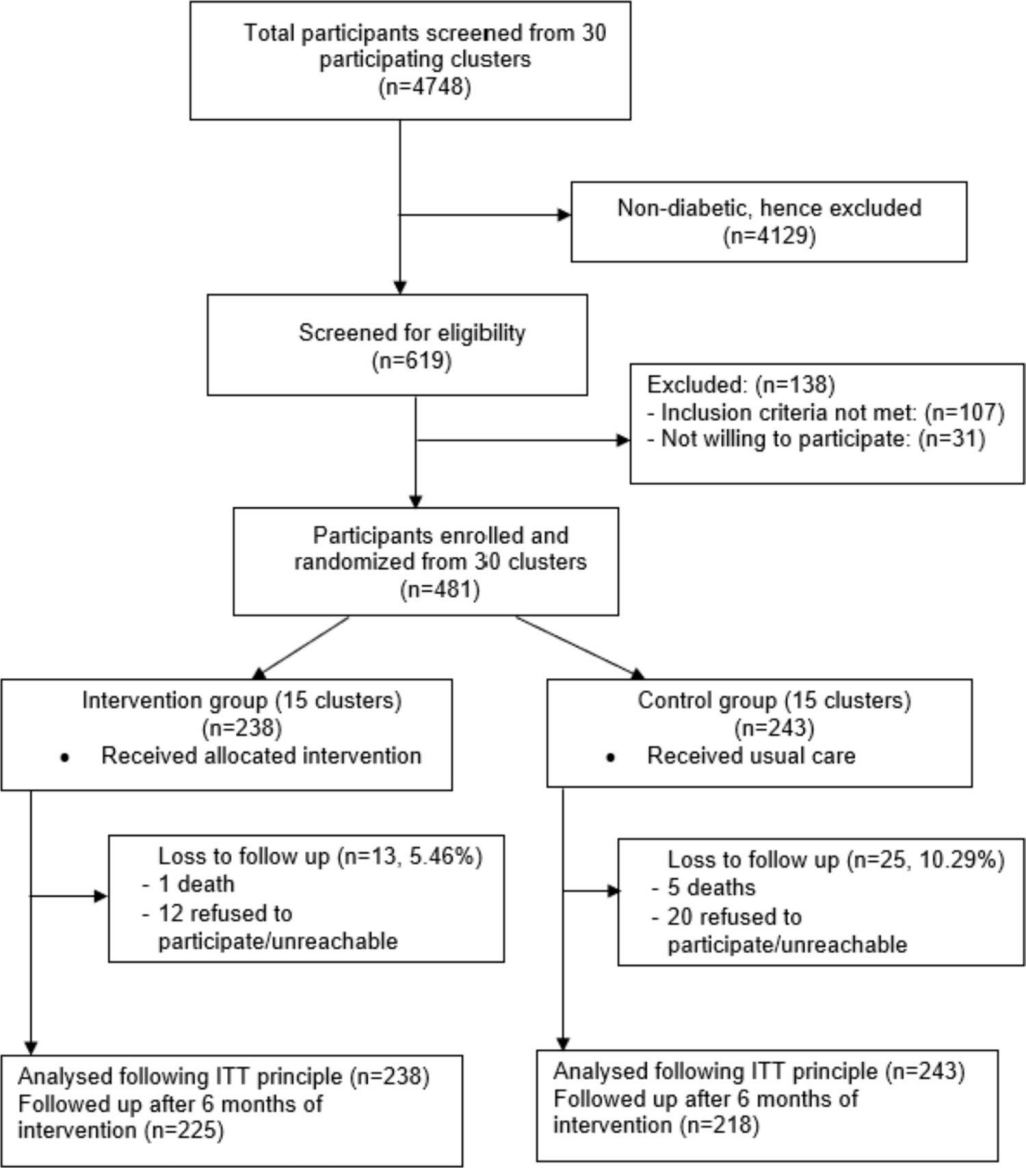
CONSORT diagram of the trial

Participants were recruited between September 2021 and February 2022. Follow-up assessments were done in October 2022. A total of 30 clusters (481 participants) were included in the study with 15 clusters (n = 238) in the intervention group and 15 clusters (n = 243) in the control group (Fig. 1). There was 13 (5.46%) loss to follow-up in the intervention arm and 25 (10.29%) in the control arm. The reasons were participants refusing to participate or being unreachable and a small number of deaths. All participants randomised to their respective groups (238 in the intervention arm and 243 in the control arm) were analysed following the ITT principle.

### Participants characteristics

The mean age of participants at baseline was 54.4 years (SD 9.4). There were no differences in baseline characteristics of participants between the intervention and control groups, as shown in Table 1.

**Table 1:** Baseline characteristics of study participants

Characteristics	Intervention group % (n) N = 238	Control group % (n) N = 243	Total % (n) N = 481	SMD
Area of residence				0.001
Rural	35.7% (85)	35.8% (87)	35.8% (172)	
Peri-urban	64.3% (153)	64.2% (156)	64.2% (309)	
Age Mean (SD)	54.3 (9.1)	54.6 (9.7)	54.4 (9.4)	0.03
Age group				
< 45	14.7% (35)	14.8% (36)	14.76% (71)	
45–59	53.4% (127)	49.8% (121)	51.56% (248)	
≥ 60	31.9% (76)	35.4% (86)	33.68% (162)	
Gender				0.09
Male	50.4% (120)	55.1% (134)	52.8% (254)	
Female	49.6% (118)	44.9% (109)	47.2% (227)	
Educational status				0.05
No formal education (Illiterate or informal)	40.3% (96)	42.4% (103)	41.4% (199)	
Below high school (below grade 10)	28.6% (68)	28.8% (70)	28.7% (138)	
Secondary level and higher (grade 10 and above)	31.1% (74)	28.8% (70)	29.9% (144)	
Marital status				0.005
Currently married	92.9% (221)	93% (226)	92.9% (447)	
Separated/Widowed/Never married	7.1% (17)	7% (17)	7.1% (34)	
Ethnicity				0.20
Brahmin/Chhetri	47.5% (113)	52.3% (127)	49.9% (240)	
Newar/Janajati	47.5% (113)	39.1% (95)	43.2% (208)	
Dalit and others	5% (12)	8.6% (21)	6.9% (33)	
Main occupation				0.14
Housewife	24.8% (59)	19.3% (47)	22% (106)	
Agriculture/Animal husbandry	42.4% (101)	43.2% (105)	42.8 (206)	
Business/Service	21.8% (52)	24.7% (60)	23.3% (112)	
Others (wage/labor/driver/retired)	10.9% (26)	12.8% (31)	11.9% (57)	
Monthly family income				0.11
≤ 10,000 NRs	26.5% (63)	25.5% (62)	26% (125)	
10,001–19999 NRs	14.3% (34)	16% (39)	15.2% (73)	
20,000–29999 NRs	17.2% (41)	20.6% (50)	18.9 (91)	
≥ 30,000 NRs	42% (100)	37.9% (92)	39.9% (192)	
Living arrangements				0.05
Living alone	2.1% (5)	2.9% (7)	2.5% (12)	
Living with family	97.9% (233)	97.1% (236)	97.5% (469)	
Diabetes-related characteristics				
Comorbidity				0.13
No	47.1% (112)	53.9% (131)	50.5% (243)	
Yes	52.9% (126)	46.1% (112)	49.5% (238)	
Duration of diabetes (years)				0.007
≤ 5	62.6% (149)	63.0% (153)	62.8% (302)	
> 5	37.4% (89)	37.0% (90)	37.2% (179)	
Taking insulin				0.02
Yes	8.4% (20)	9.1% (22)	8.7% (42)	
No	91.6% (218)	90.9% (221)	91.3% (439)	
Taking OHAs				0.006
Yes	89.9% (214)	90.1% (219)	90.0% (433)	
No	10.1% (24)	9.9% (24)	10.0% (48)	
Consumption of tobacco/products				0.17
Yes	23.9% (57)	31.7% (77)	27.9% (134)	
No	76.1% (181)	68.3% (166)	72.1% (347)	
Current alcohol consumption				0.09
Yes	20.6% (49)	24.7% (60)	22.7% (109)	
No	79.4% (189)	75.3% (183)	77.3% (372)	
Moderate to vigorous physical activity for at least 150 min per week				0.05
Yes	81.1% (193)	79.0% (192)	80.0% (385)	
No	18.9% (45)	21.0% (51)	20.0% (96)	
Hypertension				0.05
Yes	46.2% (110)	43.6% (106)	44.9% (216)	
No	53.8% (128)	56.4% (137)	55.1% (265)	
Depressive symptoms				0.01
Yes	25.2% (60)	25.9% (63)	25.6% (123)	
No	74.8% (178)	74.1% (180)	74.4% (358)	
HbA1c Mean (SD)	8.17 (2.10)	8.19 (2.01)	8.18 (2.05)	0.008
HbA1c				
< 7 (Controlled HbA1c)	33.6% (80)	32.9% (80)	33.3% (160)	
≥ 7 (Uncontrolled HbA1c)	66.4% (158)	67.1% (163)	66.7% (321)	
BMI Mean (SD)	26.75 (4.41)	27.09 (5.88)	26.93 (5.20)	0.06
BMI				
Underweight	0.8% (2)	2.1% (5)	1.5% (7)	
Normal	18.1% (43)	12.3% (30)	15.2% (73)	
Overweight	16.4% (39)	24.3% (59)	20.4% (98)	
Obese	64.7% (154)	61.3% (149)	63.0% (303)	

*SMD* Standardized mean difference, *OHA* Oral hypoglycaemic agent, *HbA1c* glycated haemoglobin, *BMI* Body mass index, *SD* Standard deviation

### Differences between baseline and follow-up health-related quality of life by study groups

No difference in the EQ5D-3L score was observed within both intervention and control groups (Table 2). The EQVAS score improved slightly at six-month follow-up in the intervention group (baseline: 72.15, 95%CI: 70.20–74.10 vs. follow up: 72.35, 95%CI: 70.15–74.54, p-value 0.89), however this improvement was not statistically significant. A statistically significant (p < 0.03) reduction in EQVAS was observed in the control group (baseline: 71.21, 95%CI: 69.17–73.25 vs. follow up: 68.02, 95%CI: 65.96–70.07). The difference in change between the groups for EQ5D-3L was 0.016 (95%CI: -0.03, 0.07), favouring the intervention group. Similarly, a difference in change between groups of 3.39 (95% CI: − 0.73, 7.50) was observed for EQVAS score, also in favour of the intervention group.

**Table 2:** Changes in HRQOL outcomes between intervention and control groups (crude estimation)

Outcome variable	Differences of HRQOL outcomes at 95% confidence interval								Between group differences
	Intervention group				Control group				
	Baseline (N = 238)	Follow-up (N = 238)	Difference (E–B)	P within group	Baseline (N = 243)	Follow-up (N = 243)	Difference (E–B)	P within group	
EQ5D-3L	0.976 (0.97, 0.98)	0.976 (0.97, 0.98)	0 (− 0.01, 0.01)	1.00	0.976 (0.96, 0.99)	0.959 (0.91, 1.01)	− 0.016 (− 0.06, 0.03)	0.51	0.016 (− 0.03, 0.07)
EQVAS	72.15 (70.20, 74.10)	72.35 (70.15, 74.54)	0.20 (− 2.73, 3.13)	0.89	71.21 (69.17, 73.25)	68.02 (65.96, 70.07)	− 3.19 (− 6.08, − 0.29)	0.03*	3.39 (− 0.73, 7.50)

Bold p-value indicates statistical significance at p < 0.05

### Intervention effect on health-related quality of life outcomes

The effect of intervention on EQ5D-3L and EQVAS using a GEE model is presented in \* MERGEFORMAT Table 3. A positive but non-significant intervention effect was seen in the EQ5D-3L index score across the study population in the adjusted model (β_1_ = 0.052, 95%CI: − 0.04, 0.14). The intervention had a positive and statistically significant effect on EQVAS score (β_1_ = 3.61, 95%CI: 0.05, 7.17) in the adjusted model.

### Association between number of sessions attended and health-related quality of life

**Fig. 2:**
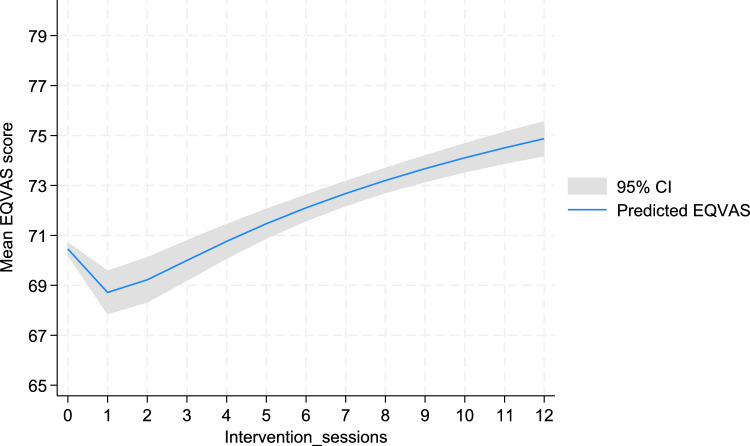
Predicted EQVAS score based on the number of intervention sessions attended

Nearly 55% of participants (n = 130) attended at least seven intervention sessions out of a total of 12. Only 16% (n = 37) attended all 12 intervention sessions while 9.6% (n = 23) did not attend any of the session (Supplementary Table S4). The average number of sessions (SD) attended was seven (SD, 4.19). The predicted EQVAS score declined slightly when intervention group participants attended only one session out of the twelve sessions compared to when they had not attended any session. However, with every additional number of sessions attended, an increase in their EQVAS score was predicted Fig. 2**.**

**Fig. 3:**
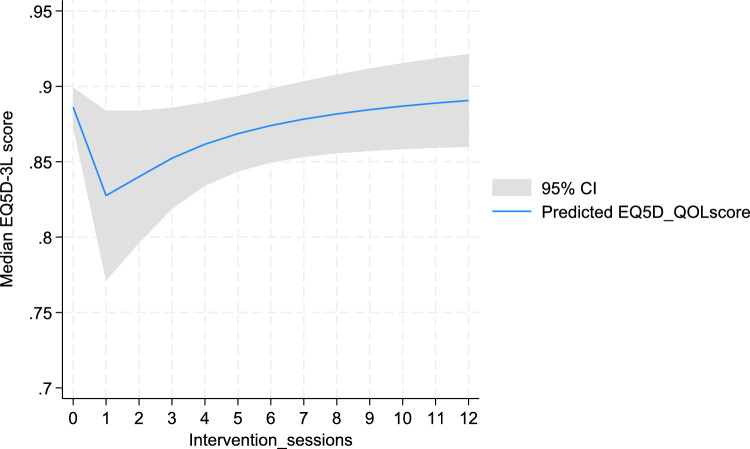
Predicted EQ5D-3L index score based on the number of intervention sessions attended

Similarly, a slight decline was observed in the predicted EQ5D-3L index score when intervention group participants attended only one session. However, an increase in the index score was predicted for every additional session attended after that as shown in Fig. 3.

Our adjusted GEE model showed a positive and statistically significant increase in EQVAS score by 0.5 per unit session attended by the study participants (β_1_ = 0.49, 95% CI: 0.01, 0.98). Further, the EQVAS score of those who attended seven or more intervention sessions was higher by four points (β_1_ = 3.86, 95% CI: -0.13, 7.85) than of those who attended six or less sessions, although this difference was not statistically significant (p = 0.06) (Supplementary Table S5). However, we did not find any statistically significant effect of number of sessions attended on the EQ5D-3L index score (β_1_ = 0.005, 95% CI: − 0.001, 0.01) ( \* MERGEFORMAT Table 3).

**Table 3:** The effect of intervention on HRQOL outcomes using GEE model

Outcome	Intervention effect			
	Unadjusted regression coefficient, β₀ (95% CI)	P-value	Adjusted regression coefficient, β_1_ (95% CI)	P-value
Overall intervention effect				
EQ5D-3L^#^	0.047 (− 0.05, 0.15)	0.35	0.052 (− 0.04, 0.14)	0.27
EQVAS^^^	3.41 (− 0.56, 7.39)	0.09	3.61 (0.05, 7.17)	**0.05**
Effect based on the number of intervention sessions attended				
EQ5D-3L^#^	0.004 (− 0.001, 0.011)	0.16	0.005 (− 0.001, 0.01)	0.13
EQVAS^^^	0.56 (0.02, 1.09)	**0.04**	0.49 (0.01, 0.98)	**0.04**

^#^Adjusted for age, residential status, gender, marital status, education, occupation, income, alcohol, physical activity, comorbidity and presence of depressive symptoms as they were statistically significant at p < 0.20 in the univariate model

^Adjusted for age, HbA1c, gender, marital status, ethnicity, education, occupation, living arrangement, income, tobacco, alcohol, physical activity, body mass index, comorbidity and presence of depressive symptoms as they were statistically significant at p < 0.20 in the univariate model

Bold p-value indicates statistical significance at p < 0.05

Further subgroup analysis suggested a negative association of HbA1c with EQVAS (β_1_ = − 0.31, 95% CI: − 1.67, 1.05) as well as with EQ5D-3L index score (β_1_ = − 0.012, 95% CI: − 0.02, 0.003) across the intervention group population suggesting that a decline in HbA1c level was associated with an improvement in HRQOL. However, these associations were not of statistical significance (results not shown in the table).

## Discussion

To our knowledge, this study is the first to examine the effect of a culturally adapted community-based health behaviour intervention for T2DM in Nepal on HRQOL, measured by EQ5D-3L index score and EQVAS score. The intervention was designed and delivered utilizing local resources and culturally adapted approaches such as traditional dietary and physical activity practices, storytelling and experience sharing, empathetic listening to cultural and health beliefs and motivational counselling. There was a minimal and statistically insignificant increase in EQVAS from baseline to six-months follow up in the intervention group. However, the reduction in EQVAS in the control group from baseline to six months follow-up was statistically significant. Deteriorated HRQOL in the control group is evident in previous trials too [21, 22]. As such, it is possible that the intervention prevented the decline in HRQOL of people with T2DM.

Our study revealed that the intervention resulted on an average increase of EQVAS score by nearly four units in the intervention group compared to the control group at six months follow up. This between-group difference concurs with the findings from previous studies [21–24]. In the studies by Rubin et al., involving structured dietary program as intervention [23], and Macdonald et al., involving structured exercise and individualised diet plans as intervention [22], participants reported a significantly higher scores in the physical component of HRQOL after attending behavioural modification intervention compared with those receiving standard care. Similarly, another study by Nicolluci et al., which involved providing diabetes counselling and physical activity to the intervention group, reported a significant between-group differences in both physical and mental domain of HRQOL assessed by using the Short-form (SF36) tool [21]. A study by Kumari et al. involved lifestyle modification counselling on balanced diet, physical activity, tobacco and alcohol cessation, stress management, and adherence to medication and medical check-ups as intervention and reported further improvement in HRQOL at 12 months follow-up than at six months follow-up among the people following lifestyle intervention compared to the people receiving usual care [25]. However, the potential differences in socio-economic and cultural contexts and intervention details such as content, duration, frequency and setting of delivery between these studies and our study should be considered while comparing findings. A noteworthy feature of our study is that it provided culturally tailored community-based intervention whereas, there is limited information on cultural adaptability of the interventions described in the previous studies [21–25]. Contrastingly, the Look Ahead Trial found that HRQOL tended to worsen over time among people with T2DM who received intensive lifestyle intervention, although initial improvements were noted [23]. This reflects the need for conducting follow-up assessments of HRQOL outcomes among study population to estimate the true and long-term effects of the intervention on their HRQOL outcomes. Further, a T2DM management program conducted in Austria, did not report any significant changes in the EQVAS or EQ5D-3L index score between the study groups [26]. This contrast with our study could be explained by the varying intervention duration and follow up period, varying use of HRQOL assessment tool and even the duration of diabetes’ diagnosis.

No significant differences were observed for EQ5D-3L index score between the groups. Similar findings were reported in a previous diabetes self-management intervention program conducted in New Zealand, where a statistically significant improvement in EQVAS by four units was observed in the intervention group compared to usual care group but no significant differences were observed between groups for the EQ5D index score [27]. The absence of improvements in the EQ5D index score compared to EQVAS could be attributed to measurement differences between the utility-based EQ5D index and self-reported EQVAS measure or the ceiling effect exhibited by EQ5D-3L index score where many baseline participants report no problems in the EQ5D dimensions, meaning the highest possible score at baseline, as evident from our baseline findings [28], which further limits the possibility to detect further improvements [29].

There is no data available on the minimal clinically important difference for EQVAS for those with T2DM or at risk of developing T2DM, hence it limits our ability to determine whether the between group mean EQVAS increases of 3.39 after six months intervention among all participants as observed in this study is of clinical relevance or not [26, 30]. Likewise, the observed difference in EQ-5D-3L index scores between the intervention and control group at six months was reported to be 0.016 (SE: 0.04) in this study, which is less than the minimal clinically important difference reported for T2DM population (0.049 to 0.077) in a Chinese study [31]. Changes in HRQOL outcome for chronic conditions like T2DM are relatively slower and may require intervention of longer duration if they are targeted towards behavioural change [8]. Unlike our short-term intervention with follow up at six months, longer term intervention of twelve months or more are commonly practised to get a more comprehensive estimates of the clinically important differences for HRQOL especially for chronic condition like T2DM [32]. Furthermore, it is recommended to use the EQ5D as an instrument for assessing HRQOL in people with T2DM in order to better understand what changes in EQVAS or EQ-5D index values translate into clinically meaningful improvements in HRQOL.

Based on our sub-group analysis, the predicted EQVAS score declined when participants attended only one session out of the twelve. However, as they attended more sessions, an improved predicted EQVAS score was observed for every unit session attended out of the twelve intervention sessions. This could be attributed to the fact that as participants attended their first session, they got introduced to T2DM, its associated challenges, and required health behaviour change to manage the condition. This newly acquired awareness may have overwhelmed them and developed a sense of fear regarding its management in them. Subsequently, this may have resulted in a temporary decline in their HRQOL. Further, participants may have realized the gap between their existing health behaviours and the behavioural change required to optimize their glucose level, which in turn may have caused them stress impacting their perceived HRQOL. This is congruent with a study which reported an optimized QOL outcomes when participants engaged in a greater number of diabetes self-management sessions with pharmacists and life coaches [33]. Similarly, a retrospective observation of a diabetes prevention program indicated a significant association of each session attendance with a 0.0042 increase in utility [34]. Increased attendance to the intervention sessions is assumed to lead to increased diabetes-related knowledge and behaviour change practice among people with T2DM, both of which are found to have a significantly positive correlation with HRQOL outcomes [7, 35]. Further, it is also very likely that participants attending more session’s exhibit greater motivation to acquire new knowledge for managing their diabetes well [36] and feel more empowered to make their own decisions regarding self-care [37]. This association could explain the significant improvement in EQVAS for every unit session attended in our intervention group participants. A follow-up qualitative study could help explain why participants perceived their HRQOL to improve after attending greater number of intervention sessions while it could also provide some answers as to why their HRQOL declined when they attended only one session.

The higher number of deaths and refusals to participate in the control group compared to the intervention group is noteworthy. The observed attrition rate of 10% in the control group is within the acceptable rate for RCTs and is likely due to the perceived lack of support among the control group participants resulting in lower motivation and disengagement [38]. Further, closer examination of the health profiles of participants who had died revealed the presence of various comorbidities such as cardiovascular disease and renal complication. As such, the higher mortality in the control group could be explained by underlying health conditions of those participants. It could also be argued that the intervention group participants may have received potential benefits in improving self-care behaviours or glycaemic control, resulting in lesser loss to follow up in the intervention group compared to control group. However, this interpretation warrants further investigation.

The poor intervention adherence, as evident from only over half of the participants attending at least seven sessions out of the twelve, should be considered while interpreting the findings of this study. The study was conducted during the COVID-19 pandemic, and the restrictions caused by it may have significantly affected participants’ attendance to intervention sessions due to fear of transmission of Covid-19, potential reluctance to attend group-based sessions and broader societal disruptions caused by the pandemic in the country. Qualitative exploration of the barriers and challenges of intervention attendance could be of value in understanding further why there were varying levels of attendance to intervention sessions.

Strengths of this study include the RCT design and the fact that it was conducted in real-world community settings. Incorporating contextual and locally tailored approaches in the intervention modules and utilizing local resources and language in delivering the intervention while strengthening local capacity building ensured the cultural appropriateness and community ownership of the intervention. The EQ-5D utility scores from this study provide a preference-based score that can be used to calculate quality-adjusted life years for future cost-effectiveness analyses of the intervention program and to inform the scalability of such program across the nation. Training of community health care workers before they delivered the intervention modules to the participants, the large sample size and the use of validated tool for assessing HRQOL of participants add to the strength of this study. However, the study has limitations too. First, the findings of this study may not be generalizable to the T2DM population of the whole country as the characteristics of the general population across the country could be different to sample characteristics. In addition, the study may be subject to volunteer bias, meaning our study participants could be more motivated for behavioural change than the general T2DM population of the country. COVID-19 restrictions impacted the delivery of our intervention at the time, causing the duration of the intervention to be shortened from twelve months to six months. This duration may have been too short to determine an actual change in the observed HRQOL outcomes of the study participants. Since HRQOL is recognised as a multidimensional concept which is difficult to change and may take long duration to change [39], short-term interventions like ours may not have immediate effects on an individual’s perception of their health or HRQOL and may not determine an actual change in their observed HRQOL outcomes. The use of EQ5D-3L instead of its superior version (EQ5D-5L) in terms of sensitivity and precision is another limitation of the study. Assessing change in HRQOL was the secondary outcome of the trial, reduction in HbA1c being the primary one. Considering the ease and feasibility of administering a shorter version of EQ5D along with many other tools administered at the same time to gather data for other secondary outcomes of the trial, EQ5D-3L was chosen instead of EQ5D-5L. Further, the original trial registration included plans for utilizing both short form-8 (SF-8) and EQ5D to assess HRQOL changes. However, only EQ5D was included in the final analyses due to very high baseline scores observed in SF-8 analysis, causing a ceiling effect that prevented us from observing meaningful change. The lack of Nepal-specific or South Asian region-specific population norms required for the SF-8 scoring, and concerns over the validity of the SF-8 added to this decision [40]. Moreover, while the baseline data were partially collected via telephone calls due to COVID-19 travel restriction at the time, the information collected via telephone calls may have been over reported compared to those collected in person for all participants at the end of the intervention.

## Conclusion

We conclude that the community-based health behaviour intervention for T2DM management had a positive effect on HRQOL of people living with T2DM in Nepal. An increase in the number of intervention sessions attended was significantly associated with higher EQVAS scores, hence, future research should emphasize delivering interventions that are engaging and well adhered too. Further studies are recommended to assess HRQOL outcomes in T2DM population using a superior, 5-level version of EQ5D. More detailed and well-designed studies with longer term intervention are required to understand what changes in EQVAS or EQ-5D index values translate to clinically meaningful improvements in HRQOL in people living with T2DM. Further, longer term assessment of change in HRQOL outcomes are needed when actual changes in HRQOL are more likely to be observed rather than limiting to immediate post-intervention assessment.

## Supplementary Information

Below is the link to the electronic supplementary material.

Supplementary file1 (DOCX 57 KB)

Supplementary file2 (DOCX 176 KB)

## Data Availability

Data that support the findings of this study will be made available by the corresponding author upon reasonable request.

## References

[1] Gyawali, B., Sharma, R., Neupane, D., Mishra, S. R., van Teijlingen, E., & Kallestrup, P. (2015). Prevalence of Type 2 diabetes in nepal: a systematic review and meta-analysis from 2000 to 2014. *Global Health Action,**8*, 29088. 26613684 10.3402/gha.v8.29088 PMC4662667

[2] Sagastume, D., Siero, I., Mertens, E., Cottam, J., Colizzi, C., & Peñalvo, J. L. (2022). The effectiveness of lifestyle interventions on Type 2 diabetes and gestational diabetes incidence and cardiometabolic outcomes: a systematic review and meta-analysis of evidence from low- and middle-income countries. *EClinicalMedicine.,**53*, 101650. 36119561 10.1016/j.eclinm.2022.101650 PMC9475282

[3] Karamanakos, G., Costa-Pinel, B., Gilis-Januszewska, A., Velickiene, D., Barrio-Torrell, F., Cos-Claramunt, X., et al. (2019). The effectiveness of a community-based, Type 2 diabetes prevention programme on health-related quality of life. The De-Plan Study. *PLoS One.,**14*(10), e0221467. 31603914 10.1371/journal.pone.0221467 PMC6788719

[4] Luscombe, F. A. (2000). Health-related quality of life measurement in Type 2 diabetes. *Value in Health,**3*(s1), 15–28. 16464206 10.1046/j.1524-4733.2000.36032.x

[5] Skovlund, S. E., Lichtenberg, T. H., Hessler, D., & Ejskjaer, N. (2019). Can the routine use of patient-reported outcome measures improve the delivery of person-centered diabetes care? A review of recent developments and a case study. *Current Diabetes Reports,**19*(9), 84. 31420754 10.1007/s11892-019-1190-x

[6] Cochran, J., & Conn, V. S. (2008). Meta-analysis of quality of life outcomes following diabetes self-management training. *The Diabetes Educator,**34*(5), 815–823. 18832286 10.1177/0145721708323640 PMC2822439

[7] Aminuddin, H. B., Jiao, N., Jiang, Y., Hong, J., & Wang, W. (2021). Effectiveness of smartphone-based self-management interventions on self-efficacy, self-care activities, health-related quality of life and clinical outcomes in patients with Type 2 diabetes: a systematic review and meta-analysis. *International Journal of Nursing Studies,**116*, 103286. 30827741 10.1016/j.ijnurstu.2019.02.003

[8] Karki, A., Vandelanotte, C., Khalesi, S., Dahal, P., & Rawal, L. B. (2023). The effect of health behavior interventions to manage Type 2 diabetes on the quality of life in low-and middle-income countries: a systematic review and meta-analysis. *PLoS ONE,**18*(10), e0293028. 37844107 10.1371/journal.pone.0293028 PMC10578590

[9] Gyawali, B., Sharma, R., Mishra, S. R., Neupane, D., Vaidya, A., Sandbæk, A., et al. (2021). Effectiveness of a female community health volunteer-delivered intervention in reducing blood glucose among adults with Type 2 Diabetes: an open-label, cluster randomized clinical trial. *JAMA Network Open,**4*(2), e2035799. 33523189 10.1001/jamanetworkopen.2020.35799 PMC7851734

[10] Sunuwar, D. R., Nayaju, S., Dhungana, R. R., Karki, K., Singh Pradhan, P. M., Poudel, P., et al. (2023). Effectiveness of a dietician-led intervention in reducing glycated haemoglobin among people with Type 2 diabetes in Nepal: a single centre, open-label, randomised controlled trial. *Lancet Reg Health Southeast Asia.,**18*, 100285. 38028163 10.1016/j.lansea.2023.100285 PMC10667281

[11] Rawal, L., Dahal, P., Paudel, G., Biswas, T., Shrestha, R., Makaju, D., et al. (2023). Community-based lifestyle intervention for diabetes (Co-Lid Study) management rural Nepal: study protocol for a clustered randomized controlled trial. *Trials,**24*(1), 441. 37403179 10.1186/s13063-023-07451-5 PMC10320880

[12] Campbell, M. K., Piaggio, G., Elbourne, D. R., & Altman, D. G. (2012). Consort 2010 statement: extension to cluster randomised trials. *BMJ: British Medical Journal.,**345*, e5661. 22951546 10.1136/bmj.e5661

[13] World Health Organization. (1999). Definition, diagnosis and classification of diabetes mellitus and its complications: Report of a WHO Consultation. Part 1, diagnosis and classification of diabetes mellitus. Geneva: *World Health Organization*.

[14] American Diabetes Association. (2014). Diagnosis and classification of diabetes mellitus. *Diabetes Care,**37*(Suppl 1), S81-90. 24357215 10.2337/dc14-S081

[15] Borelli, M. R., Riden, H. E., Bang, H., & Schenker, M. B. (2018). Protocol for a cluster randomized controlled trial to study the effectiveness of an obesity and diabetes intervention (Pasos) in an immigrant farmworker population. *BMC Public Health*. 10.1186/s12889-018-5560-0 29986676 10.1186/s12889-018-5560-0 PMC6038353

[16] Klein, H. A., Jackson, S. M., Street, K., Whitacre, J. C., & Klein, G. (2013). Diabetes self-management education: miles to go. *Nutrition Research and Practice,**2013*, 581012. 10.1155/2013/581012 PMC3616351 23577243

[17] Kandel, N., & Lamichhane, J. (2019). Female health volunteers of Nepal: the backbone of health care. *Lancet,**393*(10171), e19–e20. 30739703 10.1016/S0140-6736(19)30207-7

[18] Balestroni, G., & Bertolotti, G. (2012). Euroqol-5D (Eq-5D): an instrument for measuring quality of life. *Monaldi Archives for Chest Disease,**78*(3), 155–159. 23614330 10.4081/monaldi.2012.121

[19] EuroQol. https://euroqol.org/support/analysis-tools/cross-walk/.

[20] Andrade, C. (2020). Mean difference, standardized mean difference (Smd), and their use in meta-analysis: as simple as it gets. *The Journal of Clinical Psychiatry.,**81*(5), 11349. 10.4088/JCP.20f13681 32965803

[21] Nicolucci, A., Haxhi, J., D’Errico, V., Sacchetti, M., Orlando, G., Cardelli, P., et al. (2022). Effect of a behavioural intervention for adoption and maintenance of a physically active lifestyle on psychological well-being and quality of life in patients with Type 2 diabetes: the Ides_2 randomized clinical trial. *Sports Medicine (Auckland, N. Z.),**52*(3), 643–654. 34599476 10.1007/s40279-021-01556-0 PMC8891112

[22] MacDonald, C. S., Nielsen, S. M., Bjørner, J., Johansen, M. Y., Christensen, R., Vaag, A., et al. (2021). One-year intensive lifestyle intervention and improvements in health-related quality of life and mental health in persons with Type 2 diabetes: a secondary analysis of the U-turn randomized controlled trial. *BMJ Open Diabetes Research & Care,**9*(1), e001840. 10.1136/bmjdrc-2020-001840 PMC7812095 33441418

[23] Rubin, R. R., Wadden, T. A., Bahnson, J. L., Blackburn, G. L., Brancati, F. L., Bray, G. A., et al. (2014). Impact of intensive lifestyle intervention on depression and health-related quality of life in Type 2 diabetes: the look ahead trial. *Diabetes Care,**37*(6), 1544–1553. 24855155 10.2337/dc13-1928 PMC4030096

[24] Wolf, A. M., Conaway, M. R., Crowther, J. Q., Hazen, K. Y., Nadler, J. L., Oneida, B., et al. (2004). Translating lifestyle intervention to practice in obese patients with Type 2 diabetes: improving control with activity and nutrition (Ican) study. *Diabetes Care,**27*(7), 1570–6. 15220230 10.2337/diacare.27.7.1570

[25] Kumari, G., Singh, V., Chhajer, B., & Jhingan, A. K. (2021). Effect of lifestyle intervention holistic approach on blood glucose levels, health-related quality of life and medical treatment cost in Type 2 diabetes mellitus patients. *Acta Scientiarum. Health Sciences,**43*, e53729.

[26] Panisch, S., Johansson, T., Flamm, M., Winkler, H., Weitgasser, R., & Sönnichsen, A. C. (2018). The impact of a disease management programme for Type 2 diabetes on health-related quality of life: multilevel analysis of a cluster-randomised controlled trial. *Diabetology and Metabolic Syndrome,**10*, 28. 29643940 10.1186/s13098-018-0330-9 PMC5892002

[27] Dobson, R., Whittaker, R., Jiang, Y., Maddison, R., Shepherd, M., McNamara, C., et al. (2018). Effectiveness of text message based, diabetes self management support programme (Sms4bg): two arm, parallel randomised controlled trial. *BMJ.,**361*, k1959. 29773539 10.1136/bmj.k1959 PMC5957049

[28] Karki, A., Vandelanotte, C., Alley, S., & Rawal, L. B. (2025). Health-related quality of life and associated factors in people with Type 2 diabetes mellitus in Nepal: baseline findings from a cluster-randomized controlled trial. *Journal of Health Psychology*. 10.1177/13591053241302877 39819046 10.1177/13591053241302877 PMC13287382

[29] Cheng, L. J., Pan, T., Chen, L. A., Cheng, J. Y., Mulhern, B., Devlin, N., et al. (2024). The ceiling effects of Eq-5d-3l and 5l in general population health surveys: a systematic review and meta-analysis. *Value in Health,**27*(7), 986–997. 38467187 10.1016/j.jval.2024.02.018

[30] Eaglehouse, Y. L., Schafer, G. L., Arena, V. C., Kramer, M. K., Miller, R. G., & Kriska, A. M. (2016). Impact of a community-based lifestyle intervention program on health-related quality of life. *Quality of Life Research,**25*(8), 1903–1912. 26896960 10.1007/s11136-016-1240-7 PMC5496447

[31] Jin, X., Liu, G. G., Gerstein, H. C., Levine, M. A. H., Guan, H., Li, H., et al. (2018). Minimally important difference and predictors of change in quality of life in Type 2 diabetes: a community-based survey in China. *Diabetes/Metabolism Research and Reviews,**34*(8), e3053. 30064154 10.1002/dmrr.3053

[32] Wing, R. R. (2010). Long-term effects of a lifestyle intervention on weight and cardiovascular risk factors in individuals with Type 2 diabetes mellitus: four-year results of the look ahead trial. *Archives of Internal Medicine,**170*(17), 1566–1575. 20876408 10.1001/archinternmed.2010.334 PMC3084497

[33] Nishita, C., Cardazone, G., Uehara, D. L., & Tom, T. (2013). Empowered diabetes management: life coaching and pharmacist counseling for employed adults with diabetes. *Health Education & Behavior,**40*(5), 581–591. 23174629 10.1177/1090198112465088

[34] McManus, E., Meacock, R., Parkinson, B., & Sutton, M. (2023). Evaluating the short-term costs and benefits of a nationwide diabetes prevention programme in England: retrospective observational study. *Applied Health Economics and Health Policy,**21*(6), 891–903. 37787972 10.1007/s40258-023-00830-8 PMC10628047

[35] Ishaq, R., Haider, S., Saleem, F., Bashir, S., Tareen, A. M., Mengal, M. A., et al. (2021). Diabetes-related knowledge, medication adherence, and health-related quality of life: a correlation analysis. *Alternative Therapies in Health and Medicine,**27*(S1), 46–53. 32663176

[36] Aziz, Z., Mathews, E., Absetz, P., Sathish, T., Oldroyd, J., Balachandran, S., et al. (2018). A group-based lifestyle intervention for diabetes prevention in low- and middle-income country: implementation evaluation of the Kerala diabetes prevention program. *Implementation Science,**13*(1), 97. 30021592 10.1186/s13012-018-0791-0 PMC6052531

[37] Lambrinou, E., Hansen, T. B., & Beulens, J. W. (2019). lifestyle factors, self-management and patient empowerment in diabetes care. *European Journal of Preventive Cardiology.,**26*(2_suppl), 55–63. 31766913 10.1177/2047487319885455

[38] Lambrinou, E., Hansen, T. B., & Beulens, J. W. J. (2021). Systematic review and meta analysis of differential attrition between active and control arms in randomized controlled trials of lifestyle interventions in chronic disease. *BMC Medical Research Methodology.,**21*(1), 122. 34126934 10.1186/s12874-021-01313-x PMC8204467

[39] Fitzpatrick, R., Fletcher, A., Gore, S., Jones, D., Spiegelhalter, D., & Cox, D. (1992). Quality of life measures in health Care. I: applications and issues in assessment. *BMJ,**305*(6861), 1074–7. 1467690 10.1136/bmj.305.6861.1074 PMC1883623

[40] Hayduk, L. A., Hoben, M., & Estabrooks, C. (2024). Evidence pointing toward invalidity of the Sf-8 physical and mental scales: a fusion validity assessment. *BMC Medical Research Methodology,**24*(1), 274. 39528919 10.1186/s12874-024-02387-z PMC11552401

