# Supplementary material for: The effect of a community-based health behaviour intervention on health-related quality of life in people with Type 2 diabetes in Nepal: a Cluster Randomized Controlled Trial
Source: Qual Life Res. 2025 Apr 7;34(12):3497–510. doi: 10.1007/s11136-025-03971-6 (PMC12689664; doi:10.1007/s11136-025-03971-6)
Supplement: Supplementary file 1 — Supplementary file1 (DOCX 57 KB) [file 11136_2025_3971_MOESM1_ESM.docx]

**The effect of a community-based health behaviour intervention on health-related quality of life in people with Type 2 diabetes in Nepal: a Cluster Randomized Controlled Trial**

Ashmita Karki^*1^, Corneel Vandelanotte^1^, M Mamun Huda^2^, Lal B. Rawal^1,3^

^1^School of Health, Medical and Applied sciences, Appleton Institute, Central Queensland University, Rockhampton, Australia

^2^Rural Health Research Institute, Charles Sturt University, Orange, NSW, Australia

^3^Translational Health Research Institute (THRI), Western Sydney University, Sydney Australia.

^*^Corresponding author: Ms. Ashmita Karki

Email: ashmita.karki@cqumail.com

Phone: +61 478478002

Postal address: Building 7, Bruce Highway, Rockhampton, Queensland 4702, Australia

ORCID: 0000-0002-2099-5768

# **Supplementary materials**

**Supplementary File S1: Intervention modules**

**Community based diabetes management in Nepal**

**Module 1: Introduction to Diabetes Mellitus**

Facilitator’s name:

Name of health facility:

Address/ place:

Date:

Start time: End time:

| **Topics/ Areas to be covered** | **Yes** | **No** | **Remarks** |
| --- | --- | --- | --- |
| A brief introduction to Diabetes Mellitus and types of Diabetes Mellitus |  |  |  |
| Risk factors of o Diabetes Mellitus |  |  |  |
| Signs and symptoms of Diabetes Mellitus |  |  |  |
| Complications due to Diabetes Mellitus |  |  |  |
| Self-management of Diabetes mellitus |  |  |  |
| Any other topic discussed (please write) ……………………………………….. |  |  |  |

**Attendance**

| **SN** | **Name of the participants/ address** | **Gender (M/ F)** | **Signature** |
| --- | --- | --- | --- |
| 1 |  |  |  |
| 2 |  |  |  |
| 3 |  |  |  |
| 4 |  |  |  |
| 5 |  |  |  |
| 6 |  |  |  |
| 7 |  |  |  |
| 8 |  |  |  |
| 9 |  |  |  |
| 10 |  |  |  |
| 11 |  |  |  |
| 12 |  |  |  |
| 13 |  |  |  |
| 14 |  |  |  |
| 15 |  |  |  |
| 16 |  |  |  |
| 17 |  |  |  |
| 18 |  |  |  |

**Community based diabetes management in Nepal**

**Module 2: Hypertension and Diabetes Mellitus**

Facilitator’s name:

Name of health facility:

Address/ place:

Date:

Start time: End time:

| **Topics/ Areas to be covered/ address** | **Yes** | **No** | **Remarks** |
| --- | --- | --- | --- |
| A brief introduction to Hypertension |  |  |  |
| Risk factors of Hypertension |  |  |  |
| Signs and symptoms of Hypertension |  |  |  |
| Complications due to Hypertension |  |  |  |
| Links between hypertension and diabetes mellitus |  |  |  |
| Early management of hypertension |  |  |  |
| Any other topic discussed (please write) ……………………………………….. |  |  |  |

**Attendance**

| **SN** | **Name of the participants** | **Gender (M/ F)** | **Signature** |
| --- | --- | --- | --- |
| 1 |  |  |  |
| 2 |  |  |  |
| 3 |  |  |  |
| 4 |  |  |  |
| 5 |  |  |  |
| 6 |  |  |  |
| 7 |  |  |  |
| 8 |  |  |  |
| 9 |  |  |  |
| 10 |  |  |  |
| 11 |  |  |  |
| 12 |  |  |  |
| 13 |  |  |  |
| 14 |  |  |  |
| 15 |  |  |  |
| 16 |  |  |  |
| 17 |  |  |  |
| 18 |  |  |  |

**Community based diabetes management in Nepal**

**Module 3: Treating and Managing Diabetes Mellitus**

Facilitator’s name:

Name of health facility: Address/ place:

Date:

Start time: End time:

| **Topics/ Areas to be covered** | **Yes** | **No** | **Remarks** |
| --- | --- | --- | --- |
| Treatment procedures/measures for Diabetes Mellitus |  |  |  |
| Importance of treating Diabetes Mellitus |  |  |  |
| Treatment or management provisions/services in Nepal |  |  |  |
| Complications of Diabetes Mellitus if delayed for treatment |  |  |  |
| Diabetes medications and its importance |  |  |  |
| Insulin and its administration techniques |  |  |  |
| Self-monitoring of blood glucose and its importance |  |  |  |
| Any other topic discussed (please write) ……………………………………….. |  |  |  |

**Attendance**

| **SN** | **Name of the participants/ address** | **Gender (M/ F)** | **Signature** |
| --- | --- | --- | --- |
| 1 |  |  |  |
| 2 |  |  |  |
| 3 |  |  |  |
| 4 |  |  |  |
| 5 |  |  |  |
| 6 |  |  |  |
| 7 |  |  |  |
| 8 |  |  |  |
| 9 |  |  |  |
| 10 |  |  |  |
| 11 |  |  |  |
| 12 |  |  |  |
| 13 |  |  |  |
| 14 |  |  |  |
| 15 |  |  |  |
| 16 |  |  |  |
| 17 |  |  |  |
| 18 |  |  |  |

**Community based diabetes management in Nepal**

**Module 4: Physical Activity**

Facilitator’s name:

Name of health facility:

Address/ place:

Date:

Start time: End time:

| **Topics/ Areas to be covered/ address** | **Yes** | **No** | **Remarks** |
| --- | --- | --- | --- |
| A brief introduction to Physical Activity |  |  |  |
| Importance/ health benefits of Physical Activity |  |  |  |
| Consequences of Physical inactivity |  |  |  |
| Recommended level of Physical Activity |  |  |  |
| Links between Physical Activity and Diabetes Mellitus |  |  |  |
| Any other topic discussed (please write) ……………………………………….. |  |  |  |

**Attendance**

| **SN** | **Name of the participants** | **Gender (M/ F)** | **Signature** |
| --- | --- | --- | --- |
| 1 |  |  |  |
| 2 |  |  |  |
| 3 |  |  |  |
| 4 |  |  |  |
| 5 |  |  |  |
| 6 |  |  |  |
| 7 |  |  |  |
| 8 |  |  |  |
| 9 |  |  |  |
| 10 |  |  |  |
| 11 |  |  |  |
| 12 |  |  |  |
| 13 |  |  |  |
| 14 |  |  |  |
| 15 |  |  |  |
| 16 |  |  |  |
| 17 |  |  |  |
| 18 |  |  |  |

**Community based diabetes management in Nepal**

**Module 5: Stress, Depression, and it’s management**

Facilitator’s name:

Name of health facility:

Address/ place:

Date:

Start time: End time:

| **Topics/ Areas to be covered/ address** | **Yes** | **No** | **Remarks** |
| --- | --- | --- | --- |
| A brief introduction to Stress and Depression |  |  |  |
| Importance of managing stress and depression |  |  |  |
| Link of stress and depression among people with type 2 diabetes mellitus |  |  |  |
| Stress and depression management among people with type 2 diabetes mellitus |  |  |  |
| Relaxation techniques |  |  |  |
| Any other topic discussed (please write) ……………………………………….. |  |  |  |

**Attendance**

| **SN** | **Name of the participants** | **Gender (M/ F)** | **Signature** |
| --- | --- | --- | --- |
| 1 |  |  |  |
| 2 |  |  |  |
| 3 |  |  |  |
| 4 |  |  |  |
| 5 |  |  |  |
| 6 |  |  |  |
| 7 |  |  |  |
| 8 |  |  |  |
| 9 |  |  |  |
| 10 |  |  |  |
| 11 |  |  |  |
| 12 |  |  |  |
| 13 |  |  |  |
| 14 |  |  |  |
| 15 |  |  |  |
| 16 |  |  |  |
| 17 |  |  |  |
| 18 |  |  |  |

**Community based diabetes management in Nepal**

**Module 6: Dietary habits**

Facilitator’s name:

Name of health facility:

Address/ place:

Date:

Start time: End time:

| **Topics/ Areas to be covered/ address** | **Yes** | **No** | **Remarks** |
| --- | --- | --- | --- |
| A brief introduction to healthy or balanced diet |  |  |  |
| Importance/ health benefits of healthy diet |  |  |  |
| Consequences of unhealthy dietary habits |  |  |  |
| Recommended dietary habits/practices for people with type 2 diabetes mellitus |  |  |  |
| Links between healthy diet and diabetes mellitus |  |  |  |
| Any other topic discussed (please write) ……………………………………….. |  |  |  |

**Attendance**

| **SN** | **Name of the participants** | **Gender (M/ F)** | **Signature** |
| --- | --- | --- | --- |
| 1 |  |  |  |
| 2 |  |  |  |
| 3 |  |  |  |
| 4 |  |  |  |
| 5 |  |  |  |
| 6 |  |  |  |
| 7 |  |  |  |
| 8 |  |  |  |
| 9 |  |  |  |
| 10 |  |  |  |
| 11 |  |  |  |
| 12 |  |  |  |
| 13 |  |  |  |
| 14 |  |  |  |
| 15 |  |  |  |
| 16 |  |  |  |
| 17 |  |  |  |
| 18 |  |  |  |

**Community based diabetes management in Nepal**

**Module 7: Tobacco and Alcohol use**

Facilitator’s name:

Name of health facility:

Address/ place:

Date:

Start time: End time:

| **Topics/ Areas to be covered/ address** | **Yes** | **No** | **Remarks** |
| --- | --- | --- | --- |
| A brief introduction on effects of tobacco and alcohol use |  |  |  |
| Importance/ health benefits of avoiding tobacco and alcohol use among people with type 2 diabetes mellitus |  |  |  |
| Consequences of tobacco use and harmful consumption of alcohol |  |  |  |
| Links between tobacco and alcohol use, and diabetes mellitus |  |  |  |
| Tips or techniques for avoiding the tobacco use and harmful consumption of alcohol |  |  |  |
| Any other topic discussed (please write) ……………………………………….. |  |  |  |

**Attendance**

| **SN** | **Name of the participants** | **Gender (M/ F)** | **Signature** |
| --- | --- | --- | --- |
| 1 |  |  |  |
| 2 |  |  |  |
| 3 |  |  |  |
| 4 |  |  |  |
| 5 |  |  |  |
| 6 |  |  |  |
| 7 |  |  |  |
| 8 |  |  |  |
| 9 |  |  |  |
| 10 |  |  |  |
| 11 |  |  |  |
| 12 |  |  |  |
| 13 |  |  |  |
| 14 |  |  |  |
| 15 |  |  |  |
| 16 |  |  |  |
| 17 |  |  |  |
| 18 |  |  |  |

**Community based diabetes management in Nepal**

**Module 8: Foot care**

Facilitator’s name:

Name of health facility:

Address/ place:

Date:

Start time: End time:

| **Topics/ Areas to be covered/ address** | **Yes** | **No** | **Remarks** |
| --- | --- | --- | --- |
| A brief introduction to foot care |  |  |  |
| Importance/ health benefits of foot care |  |  |  |
| Links between foot care and diabetes mellitus |  |  |  |
| Consequences of neglecting foot care or foot inspection |  |  |  |
| Recommended foot care practice for people with type 2 diabetes mellitus |  |  |  |
| Any other topic discussed (please write) ……………………………………….. |  |  |  |

**Attendance**

| **SN** | **Name of the participants** | **Gender (M/ F)** | **Signature** |
| --- | --- | --- | --- |
| 1 |  |  |  |
| 2 |  |  |  |
| 3 |  |  |  |
| 4 |  |  |  |
| 5 |  |  |  |
| 6 |  |  |  |
| 7 |  |  |  |
| 8 |  |  |  |
| 9 |  |  |  |
| 10 |  |  |  |
| 11 |  |  |  |
| 12 |  |  |  |
| 13 |  |  |  |
| 14 |  |  |  |
| 15 |  |  |  |
| 16 |  |  |  |
| 17 |  |  |  |
| 18 |  |  |  |

**Community based diabetes management in Nepal**

**Module 9: Oral (dental) care**

Facilitator’s name:

Name of health facility:

Address/ place:

Date:

Start time: End time:

| **Topics/ Areas to be covered/ address** | **Yes** | **No** | **Remarks** |
| --- | --- | --- | --- |
| A brief introduction to oral health |  |  |  |
| Importance/ health benefits of oral care or oral hygiene |  |  |  |
| Oral health problems and their signs and symptoms |  |  |  |
| Links between oral health and diabetes mellitus |  |  |  |
| Consequences of neglecting oral care or hygiene |  |  |  |
| Recommended oral care or hygiene behaviors for people with type 2 diabetes mellitus |  |  |  |
| Any other topic discussed (please write) ……………………………………….. |  |  |  |

**Attendance**

| **SN** | **Name of the participants** | **Gender (M/ F)** | **Signature** |
| --- | --- | --- | --- |
| 1 |  |  |  |
| 2 |  |  |  |
| 3 |  |  |  |
| 4 |  |  |  |
| 5 |  |  |  |
| 6 |  |  |  |
| 7 |  |  |  |
| 8 |  |  |  |
| 9 |  |  |  |
| 10 |  |  |  |
| 11 |  |  |  |
| 12 |  |  |  |
| 13 |  |  |  |
| 14 |  |  |  |
| 15 |  |  |  |
| 16 |  |  |  |
| 17 |  |  |  |
| 18 |  |  |  |

**Community based diabetes management in Nepal**

**Module 10: Sexual health**

Facilitator’s name:

Name of health facility:

Address/ place:

Date:

Start time: End time:

| **Topics/ Areas to be covered/ address** | **Yes** | **No** | **Remarks** |
| --- | --- | --- | --- |
| A brief introduction to Sexual or reproductive health |  |  |  |
| Importance of sexual or reproductive health |  |  |  |
| Sexual or reproductive health problems and their signs and symptoms |  |  |  |
| Prevention and management of the sexual or reproductive health related problems |  |  |  |
| Any other topic discussed (please write) ……………………………………….. |  |  |  |

**Attendance**

| **SN** | **Name of the participants** | **Gender (M/ F)** | **Signature** |
| --- | --- | --- | --- |
| 1 |  |  |  |
| 2 |  |  |  |
| 3 |  |  |  |
| 4 |  |  |  |
| 5 |  |  |  |
| 6 |  |  |  |
| 7 |  |  |  |
| 8 |  |  |  |
| 9 |  |  |  |
| 10 |  |  |  |
| 11 |  |  |  |
| 12 |  |  |  |
| 13 |  |  |  |
| 14 |  |  |  |
| 15 |  |  |  |
| 16 |  |  |  |
| 17 |  |  |  |
| 18 |  |  |  |

**Community based diabetes management in Nepal**

**Module 11: Safe travel tips for managing the diabetes mellitus**

Facilitator’s name:

Name of health facility:

Address/ place:

Date:

Start time: End time:

| **Topics/ Areas to be covered/ address** | **Yes** | **No** | **Remarks** |
| --- | --- | --- | --- |
| A brief introduction to safe and healthy travel |  |  |  |
| Best possible ways to adhere medication during travel |  |  |  |
| Complying with the self-care management behavior during the travel |  |  |  |
| Seeking the medical care if away from home |  |  |  |
| Any other topic discussed (please write) ……………………………………….. |  |  |  |

**Attendance**

| **SN** | **Name of the participants** | **Gender (M/ F)** | **Signature** |
| --- | --- | --- | --- |
| 1 |  |  |  |
| 2 |  |  |  |
| 3 |  |  |  |
| 4 |  |  |  |
| 5 |  |  |  |
| 6 |  |  |  |
| 7 |  |  |  |
| 8 |  |  |  |
| 9 |  |  |  |
| 10 |  |  |  |
| 11 |  |  |  |
| 12 |  |  |  |
| 13 |  |  |  |
| 14 |  |  |  |
| 15 |  |  |  |
| 16 |  |  |  |
| 17 |  |  |  |
| 18 |  |  |  |

**Community based diabetes management in Nepal**

**Module 12: Social and emotional support/ Healthcare utilization**

Facilitator’s name:

Name of health facility:

Address/ place: Date:

Start time: End time:

| **Topics/ Areas to be covered/ address** | **Yes** | **No** | **Remarks** |
| --- | --- | --- | --- |
| A brief introduction to social and emotional support |  |  |  |
| Importance of social and emotional support to people with type 2 diabetes mellitus |  |  |  |
| Ways of providing social and emotional support for people with type 2 diabetes mellitus |  |  |  |
| Health Insurance and its importance |  |  |  |
| A brief introduction to healthcare utilization and its importance |  |  |  |
| Recommended visit to the healthcare facility (specialists) for managing or treating the type 2 diabetes mellitus |  |  |  |
| Any other topic discussed (please write) ……………………………………….. |  |  |  |

**Attendance**

| **SN** | **Name of the participants** | | **Gender (M/ F)** | **Signature** |
| --- | --- | --- | --- | --- |
| 1 |  | |  |  |
| 2 |  | |  |  |
| 3 |  | |  |  |
| 4 |  | |  |  |
| 5 |  | |  |  |
| 6 |  | |  |  |
| 7 |  | |  |  |
| 8 |  | |  |  |
| 9 |  | |  |  |
| 10 |  | |  |  |
| 11 |  | |  |  |
| 12 |  | |  |  |
| 13 |  | |  |  |
| 14 |  | |  |  |
| 15 |  | |  |  |
| 16 |  | |  |  |
| 17 |  | |  |  |
| 18 |  | |  |  |
| Cultural practices adopted in the intervention | | The intervention was guided by culturally tailored practices throughout delivery. Group-based sessions on local practices such as cooking, group exercise, yoga, were conducted to complement the intervention modules and enhance participants’ locally acquired skills related to meal preparation and group exercises. The cultural practices included culturally tailored dietary education respecting traditional eating habits and local recipes; meal preparation using local produces; behavioural education on traditional physical activities such as farming and household chores; culturally relevant approaches such as use of local language, narratives, metaphors, storytelling and knowledge sharing; provision of pictorial book on diabetes management and prevention of complication in Nepali language; empathetic listening and counselling; and family and social support. Furthermore, the intervention program was delivered by trained community health workers (CHWs) and peer supporters, who later facilitated the group-based intervention to the intervention group participants. Culturally adapted practices as described above were emphasized in the training provided to CHWs and peer supporters. CHWs are the primary point of contact for health-related issues among the local population; therefore, the Training of Trainers model was adopted for local capacity building of CHWs in the two districts (Health Assistant, Auxiliary Nurse Mid-wife or Auxiliary Health Worker) in delivering the intervention sessions. This also helped foster the cultural appropriateness of the intervention. Additionally, to ensure regular contact between CHWs and participants and maintenance of self-care behaviours learnt in the sessions, CHWs conducted fortnightly telephone calls for the first three months and monthly calls for the rest of the months, along with online pictorial and audio-visual messages on lifestyle intervention in Nepali language sent to the participants’ phones. Further, the reminders for monthly peer support meetings were sent (via phone calls and message) to participants. | | |

**Supplementary Table S3: Measurement of independent variables in the study**

| **Variables** | **Tools used** | **Scoring** | **Psychometric properties** |
| --- | --- | --- | --- |
| **Physical activity** | Global Physical Activity Questionnaire (GPAQ) | Those who did moderate to vigorous PA for 150 minutes per week were considered physically active [1]. | ICC of 0.8 and spearman’s Rho of 0.6) in South Asian setting [2]. |
| **Diabetes medication adherence** | Eight-item Morisky Medication Adherence Scale (MMAS) | Those scoring <6, 6 to <8, and 8 on the scale were considered to have low, medium and high medication adherence respectively [3]. | Cronbach’s alpha of 0.61 and ICC of 0.83) in Thailand [4]. |
| **Blood pressure** | Measurement taken by a healthcare professional | Blood pressure measurement of 130/80 mmHg or higher was established as hypertension [5]. |  |
| **Glycated haemoglobin (HbA1c)** | A sensitive point-of-care testing (POCT) analyzer (SDA1c Care) | HbA1c level of 7% or more was considered uncontrolled HbA1c [6]. | A sensitivity of 95% and Lin’s concordance correlation coefficient of 0.88 [7] in South Asian settings |
| **Body mass index (BMI)** | BMI was calculated following the World Health Organization BMI criteria for Asian classification [8]. | BMI of <18.5 kg/m2 were classified as “underweight”, 18.5- 22.9 as “normal weight”, 23-24.9 as “overweight” and ≥ 25 as “obese” [8]. |  |

**Supplementary Table S4: Total number of intervention sessions attended by participants in the intervention group**

| **Number of intervention sessions attended** | **Frequency** | **Percentage** |
| --- | --- | --- |
| Mean sessions attended (SD) | 6.63 (4.19) | |
| 0 | 23 | 9.66 |
| 1 | 27 | 11.34 |
| 2 | 13 | 5.46 |
| 3 | 5 | 2.10 |
| 4 | 8 | 3.36 |
| 5 | 11 | 4.62 |
| 6 | 21 | 8.82 |
| 7 | 16 | 6.72 |
| 8 | 19 | 7.98 |
| 9 | 16 | 6.72 |
| 10 | 17 | 7.14 |
| 11 | 25 | 10.50 |
| 12 | 37 | 15.55 |
| Total = 12 | 238 | 100 |

**Supplementary Table S5: Intervention effect based on the number of intervention sessions attended**

| **HRQOL outcomes** | **Intervention effect** | | | |
| --- | --- | --- | --- | --- |
|  | **Unadjusted Regression coefficient (95% CI)** | **P-value** | **Adjusted Regression coefficient (95% CI)** | **P-value** |
| **EQ5D-3L^#^** |  |  |  |  |
| Intervention sessions (7 or more) | 0.029 (-0.026, 0.085) | 0.30 | 0.020 (-0.027, 0.067) | 0.40 |
| Intervention sessions (6 or less) | Ref |  | Ref |  |
| **EQVAS^^^** |  |  |  |  |
| Intervention sessions (7 or more) | 4.21 (-0.13, 8.56) | 0.057 | 3.86 (-0.13, 7.85) | 0.058 |
| Intervention sessions (6 or less) | Ref |  | Ref |  |

^#^Adjusted for Age, residential status, gender, marital status, education, occupation, income, alcohol, physical activity, comorbidity and presence of depressive symptoms as they were statistically significant at p<0.20 in the univariate model.

^Adjusted for Age, HbA1c, Gender, Marital status, Ethnicity, Education, Occupation, living arrangement, Income, Tobacco, Alcohol, PA, BMI, Comorbidity and presence of depressive symptoms as they were statistically significant at p<0.20 in the univariate model

**References:**

1. World Health Organization. Global Physical Activity Questionnaire (Gpaq) Analysis Guide. *Geneva: World Health Organization*. 2012:1-22.

2. Misra P, Upadhyay RP, Krishnan A, Sharma N, Kapoor SK. A Community Based Study to Test the Reliability and Validity of Physical Activity Measurement Techniques. *Int J Prev Med*. 2014;5(8):952-9.

3. Krousel-Wood M, Islam T, Webber LS, Re RN, Morisky DE, Muntner P. New Medication Adherence Scale Versus Pharmacy Fill Rates in Seniors with Hypertension. *Am J Manag Care*. 2009;15(1):59-66.

4. Sakthong P, Chabunthom R, Charoenvisuthiwongs R. Psychometric Properties of the Thai Version of the 8-Item Morisky Medication Adherence Scale in Patients with Type 2 Diabetes. *Ann Pharmacother*. 2009;43(5):950-7.

5. Organization WH, Group ISoHW. World Health Organization (Who)/International Society of Hypertension (Ish) Statement on Management of Hypertension. *Journal of hypertension*. 2003;21(11):1983-92.

6. American Diabetes Association. Diagnosis and Classification of Diabetes Mellitus. *Diabetes Care*. 2014;37 Suppl 1:S81-90.

7. Khadanga S, Singh G, Pakhare AP, Joshi R. Diagnostic Accuracy of Point-of-Care Tests Measuring Glycosylated Haemoglobin (Hba1c) for Glycemic Control: A Field Study in India. *Cureus*. 2021;13(9):e17920.

8. World Health Organization. The Asia-Pacific Perspective: Redefining Obesity and Its Treatment. 2000.
