# Supplementary material for: The effect of a community-based health behaviour intervention on health-related quality of life in people with Type 2 diabetes in Nepal: a Cluster Randomized Controlled Trial
Source: Qual Life Res. 2025 Apr 7;34(12):3497–510. doi: 10.1007/s11136-025-03971-6 (PMC12689664; doi:10.1007/s11136-025-03971-6)
Supplement: Supplementary file 2 — Supplementary file2 (DOCX 176 KB) [file 11136_2025_3971_MOESM2_ESM.docx]

**The effect of a community-based health behaviour intervention on health-related quality of life in people with Type 2 diabetes in Nepal: a Cluster Randomized Controlled Trial**

Ashmita Karki^*1^, Corneel Vandelanotte^1^, M Mamun Huda^2^, Lal B. Rawal^1,3^

^1^School of Health, Medical and Applied sciences, Appleton Institute, Central Queensland University, Rockhampton, Australia

^2^Rural Health Research Institute, Charles Sturt University, Orange, NSW, Australia

^3^Translational Health Research Institute (THRI), Western Sydney University, Sydney Australia.

^*^Corresponding author: Ms. Ashmita Karki

Email: ashmita.karki@cqumail.com

Phone: +61 478478002

Postal address: Building 7, Bruce Highway, Rockhampton, Queensland 4702, Australia

ORCID: 0000-0002-2099-5768

**Supplementary materials**

**Supplementary File S2: Comprehensive training guide for Community health workers**

**Community based lifestyle intervention for management of Type 2 diabetes in Nepal, Intervention Guide for training Community Health Workers, 2020**

**Project partners:**

Tokyo Women’s Medical University, Japan Dhulikhel Hospital, Kathmandu University, Nepal Central Queensland University, Australia

**Project funding:**

Japan Agency for Medical Research and Development (AMED) Japan, under the Global Alliance for Chronic Disease (GACD) Prevention and Control global funding Scheme

## HOW TO USE THIS MANUAL?

***Intervention guide for Diabetes education for Community Health Workers*** will be used by trainers of diabetes training for CHWs. The trainers will be team members of the project and experts from Dhulikhel Hospital working in the field of diabetes such as Physicians, nurses, nutritionists, physiotherapists, pharmacists, paramedics working. The manual will guide the trainer to conduct the training for a total 3 working days. The manual includes instructions for the trainer on how to conduct the sessions. The detail of the contents is also enclosed in the trainee‘s manual. Elaboration of different teaching learning methods and audio visual aids is kept for both the trainers and trainees at the beginning of the manual. The 3 day schedule comprises a total of 12 modules. Twelve modules includes as follows:

Module 1: Introduction of diabetes, Risk factors, symptoms and investigations of diabetes mellitus, Hypoglycemia, Complication of DM, Treatment and management of diabetes mellitus.

Module 2: Blood Pressure and Diabetes Module 3: Medication

Module 4: Physical Activity

Module 5: Depression, Stress and time management Module 6: Dietary habits

Module 7: Behavior Change: Alcohol consumption, Smoking Module 8: Foot care

Module 9: Oral Health Module 10: Sexual Health

Module 11: Travel tips and Sick Days

Module 12: Social and Emotional Supports / Meet to the health Professional

The training program also includes class assignments. There will be pre-test and post-test of the whole training program. There will be 10 questions for both pre- test and final evaluation. Participants who participate in 3 days training will be certified by Dhulikhel Hospital Kathmandu University Hospital.

## INTRODUCTION

Diabetes is one of the prevailing conditions that is considered under Non-communicable diseases [NCD] (Cardiovascular disease [CVD], diabetes, chronic pulmonary obstructive disease, asthma and cancer). The world is moving to prevent and control these epidemics. The Sustainable Development Goals (SDG) has also addressed health as one of its important components out of their 17 components. Further, global health targets (2015-2025) clearly define its nine targets in order to prevent and control NCD globally.

The World Health Organization (WHO) has also provided Package of Essential Non communicable disease Intervention (PEN) since 2016. Nepal Government has published a training manual for the PEN intervention at Primary Health Service Setting. Being aligned with PEN, Dhulikhel Hospital-Kathmandu University Hospital (DH-KUH) has committed to work on ‘Diabetes Training Manual for CHWs‘under project titled ***“A cluster randomized trial of a community-based lifestyle intervention for diabetes management in Kavrepalanchowk and Nuwakot district of Nepal”.***

The training will have two books, a trainee‘s book (Participant handbook) and a trainer‘s guide. The training is designed especially for CHWs starting from a proficiency level.

**COURSE DESCRIPTION**

The course is designed to impart theoretical and practical knowledge and skill on diabetes care among CHWs of different health centers starting from proficiency level. The course is designed for 3 working days including both theoretical and practical sessions. There are twelve modules within the course which has been described as follows:

Module 1: Introduction of diabetes, Risk factors, symptoms and investigations of diabetes mellitus, Hypoglycemia, Complication of DM, Treatment and management of diabetes mellitus.

Module 2: Blood Pressure and Diabetes Module 3: Medication

Module 4: Physical Activity

Module 5: Depression, Stress and time management Module 6: Dietary habits

Module 7: Behavior Change: Alcohol consumption, Smoking Module 8: Foot care

Module 9: Oral Health Module 10: Sexual Health

Module 11: Travel tips and Sick Days

Module 12: Social and Emotional Supports / Meet to the health Professional

# TWELVE MODULE

**MODULE 1:** INTRODUCTION OF DIABETES, RISK FACTORS, SYMPTOMS AND INVESTIGATIONS OF DIABETES MELLITUS, HYPOGLYCEMIA, COMPLICATION OF DM, TREATMENT AND MANAGEMENT OF DIABETES MELLITUS.

The course begins with an introduction and thereby a discussion of the concept of diabetes, its epidemiological situation and burden at the global, regional and national levels. Participants will also discuss normal physiology of glucose metabolism and pathogenesis of diabetes along with its classification. This module covers the risk factors of diabetes mellitus, its symptoms and required investigations to confirm the diagnosis and the investigations for regular checkup to identify the good control of blood sugar among diabetes patients. Also this consists of both acute and chronic complications of diabetes mellitus along with their proper management. Acute complication includes Diabetes Ketoacidosis (DKA), Hyperosmolar Hyperglycemic State (HHS) and Hypoglycemia. Macrovascular complications include Myocardial Infarction (MI), Stroke, and peripheral artery disease and microvascular complications include Diabetic retinopathy, Diabetic nephropathy, and Diabetic neuropathy.

**MODULE 2:** BLOOD PRESSURE AND DIABETES

This module gives the concept of good management of blood pressure to decrease the risk of stroke, heart disease, kidney disease, eye disease and nerve damage in people with diabetes. Participants will also discuss the interval of regular checkup of blood pressure and causes of high blood pressure and steps to reduce.

**MODULE 3:** MEDICATION

This module includes classification of antidiabetic agents (medicines) and elaborate the medication treatment of DM.

**MODULE 4:** PHYSICAL ACTIVITY

This module explains exercise regime such as type of exercise and its benefits. It also comprises exercise perception in patients with diabetes and recommend exercise in person with Diabetes and elaborate its potential adverse effects. It offers several suggestions for ways to be physically active indoors to stay motivated.

**MODULE 5:** DEPRESSION, STRESS AND TIME MANAGEMENT

This module explains depression, stress and management of time to reduce the disease. It also explores the causes of depression and stress, dealing with them, treatment and ways to improve time-management.

**MODULE 6:** DIETARY HABITS

This module includes Healthy eating to manage the blood glucose levels, to reduce blood fats (cholesterol and triglycerides) and maintain a healthy weight. It also clarified on recognizing high- fat and high-calorie foods and then reducing the fat and calories in our diet in order to lose weight and prevent complications.

**MODULE 7:** BEHAVIOR CHANGE: ALCOHOL CONSUMPTION, SMOKING

This module discusses the health consequences of alcohol and tobacco consumption and ways to reduce intake by substituting for healthier alternatives. Also it discusses the triggers for alcohol and tobacco consumption and how to react differently to these triggers.

**MODULE 8:** FOOT CARE

This module explains diabetes and the need of regular checkup of foot as well as caring of foot to prevent the development of diabetes complications, including problems with feet such as diabetic foot ulcer.

**MODULE 9:** ORAL HEALTH

This module despite the link between diabetes and oral health. Oral health problems in diabetes and its management.

**MODULE 10:** SEXUAL HEALTH

This module discusses causes of sexual problems with diabetes, effect on male and female sexual health due to diabetes and management of sexual health problems.

**MODULE 11:** TRAVEL TIPS AND SICK DAYS

This module includes the planning tips, ways of staying well during travel by the diabetes patient. Also the management of blood sugar when sick.

**MODULE 12:** SOCIAL AND EMOTIONAL SUPPORTS / MEET TO THE HEALTH PROFESSIONAL

This module includes social and emotional support and its impact on the physical and mental health of diabetic patients. Also help to enhance opportunities for people with diabetes to talk about their emotional well-being with their health professionals and, if problems are present, to identify and address them.

## COURSE GOAL

The goal of this course is to train CHWs and develop diabetes educators with certification from Dhulikhel Hospital Kathmandu University hospital.

**COURSE OBJECTIVES**

The objectives of the training are as follows:

- To deliver theoretical knowledge on diabetes and practical skills related to diabetes care to the trainees
- To build the capacity of CHWs as well as their engagement in the intervention implementation process
- To qualified trainees to use ‘facilitators’ handbook in the intervention implementation phase and conduct diabetes-self management intervention sessions to the T2DM patients.
- To deliver practical skills for follow up to the participants to ensure adoption and maintenance of healthy behavior such as regular PA, healthy food, no smoking, and use of health care services when needed and use of telephone calls (via automated phone calls and message) for the monthly for peer support meetings.
- To empower trainees to organize local practices such as Cooking club, “create group for physical activity”, “Yoga sessions” on a regular basis.

## COMPETENCY FOCUSED

### The training course is intended to

↑ Increase and build Knowledge

↑ Enhance the application of skills and

↑ Attitude and Practices of CHWs

These are the expected competencies to be acquired by trainees including their ability to do the following:

| 1 | Reviewing the available health registries from the health centers to found the diabetes patients |
| --- | --- |
| 2 | Conduct diabetes screening camps in the community for the recruitment of participants |
| 3 | Measure weight, height, waist circumference and calculate Body Mass Index (BMI) |
| 4 | Able to measure blood glucose using glucometer and interpret results |
| 5 | Able to measure blood pressure (BP) using both manual BP device and digital BP set and interpret results |
| 6 | Conduct group based intervention sessions of 12 months for peer supporters and participants |
| 7 | Regular contact with participants and follow up (conducting a meeting with participants once a month) |
| 8 | Organize local practices such as cooking club, create group for physical activity, Yoga sessions etc |

| 9 | Record, collect, generate and report services data related to diabetes |
| --- | --- |

## ARRANGEMENTS

**TEACHING LEARNING TECHNIQUES**

Participants are CHWs with cumulative experiential knowledge and practice in diabetes care. Teaching–learning techniques are designed according to the background of the participants. Overall, the training sessions are learner-centric, and made practical using adult learning techniques promoting problem solving critical thinking and collaborative learning.

### Learning will be enhanced by using effective methods such as:

- Presentation
- Case studies
- Session end review questions
- Group discussion
- Role play
- Individual and group practicum
- Videos
- Review of learning
- Action planning

## PARTICIPANTS WILL BE ENCOURAGED

- To stay engaged in learning
- To ask questions
- To share experiences
- To learn from discussions (learning from each other)
- To make useful notes
- To minimize outside distractions

## TRAINING DESIGN AND IMPLEMENTATION

Training materials should be prepared before the training commences. The following training documents and instruments should be provided during the training:

### Training multimedia equipment (Must needed)

1. LCD Projector with laptop
2. Slider with pointer
3. Speaker/Sound system
4. Multiplug

### Training documents

1. Trainer‘s guide
2. Trainee‘s manual
3. Video
4. Counselling and patient education aids (flipchart, flex)
5. Sheet (Recording and reporting sheet)
6. Pre-test and post-test questions
7. Other necessary training aids

### Stationary

1. Notebook with pen
2. Meta card
3. News print, cardboard paper
4. Marker (Different Colour)
5. Pencil with eraser, sharpener
6. Masking tape
7. Register
8. White board

## CLINICAL LOGISTICS

| 1 | Glucometer set | 8 |
| --- | --- | --- |
| 2 | Test strip | 8 |
| 3 | Cotton with spirit swap | Small packet |
| 4 | lancet | 16 |
| 5 | Weighing scale | 2 |
| 6 | Stadiometer (Potable) | 4 |
| 7 | Non-elastic, non-tensile measuring tape | 4 |
| 8 | Blood pressure machine | 4 |
| 9 | Disposable plastic | 4 |
| 10 | Loose Gloves | 1 pack |
| 11 | Zipper bag | 4 |
| 12 | Safety box | 4 |
| 13 | Insulin therapy set | 8 |
| 14 | HbA1c Analyzer | 1 |
| 15 | reagent | 1 |

**TRAINING DURATION**

In general, training for trainees is designed for total working 3 days. The details about the schedule is attached under the section of schedule.

- - The training course follows a very strict time table (duration)
  - It is therefore essential that the sessions begin and end at the allocated time

## TRAINING FACILITIES OF VENUE

This is a participatory training, requiring trainees to participate in role-plays, group discussion & case based learning activities. It is therefore essential and preferred to use a room that does not resemble a lecture room. The room should be large enough to allow the anticipated number of trainees to be seated in small table groups (usually not more than five per table) or U shaped, and should have enough space for participants to engage in learning activities that require individuals to move around the room.

It is further recommended that training venues have an adequate number of toilet facilities, along with temperature maintaining and lighting to ensure a comfortable atmosphere for training. A backup power supply is highly recommended.

- - It tends to contribute to the general satisfaction of trainees and allows them to focus on the material being learned.
  -
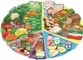
It also creates flexibility within the program. There should be a need to shorten breaks or complete work within a break.

## REFRESHMENTS RECOMMENDED

- - Timely consider providing meals to the trainees at the training venue.
  - Food should be provided on time as per schedule. The food composition should considered as heart healthy food category


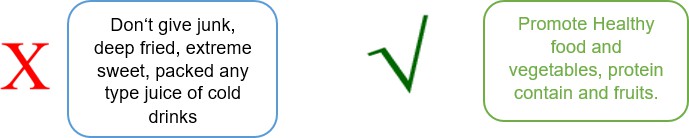


Also explain about the stretching exercises to be done at the end of the sessions & to be managed as needed

## ADMINISTRATIVE MANAGEMENT OF TRAINEE AND TRAINER THEMSELVES

- - All trainees should fill up the form for registration.
  - A training bag consisting of the trainee's manual, notebook, pen and schedule should be provided to the trainees.
  - Ensure if any official letters are required from the trainees or are needed to be handed over to the trainees.
  - There is no provision of travelling allowance and daily allowance to the trainees.

### LEGISLATION OF DIABETES TRAINING FOR CHWs AND TOT TRAINING OF TRAINEE

Trainees who would be trained from a team of training will be considered as trainers for diabetes education and they will in turn conduct group based sessions for T2DM and peer supporters. The training team will include physicians, nutritionists, physiotherapists, Pharmacist, Diabetes educator and trained nurses. The trained CHWs who have participated and involved in prior training on diabetes and possess good understanding of diabetes education, must be included in group based sessions during the intervention period.

**Criteria of participants of Training Numbe**r: Only 32 Participants

**Criteria:** working experiences as in-service CHWs (HA, AHW and ANM) working in different health centers such as government health facilities (PHC, HP, UHC) and outreach centers of Dhulikhel Hospital.

### Qualifications

- - For HA: Passed certificate of General Medicine of 3 years program affiliated to CTEVT
  - For AHW:Passed AHW or [CMA course](http://edusanjal.com/course/tslc-in-community-medicine-assistant-cma-ctevt) from recognized institution
  - For ANM: ANM graduates, at least an 18 month course of ANM

### Ratio of trainer and trainee for training of service provider

At least 5 trainer for 32 trainee Clinical (At least 1 to 2)

Nutritionist (1)

Physiotherapist (1)

Pharmacist (1)

Dentist (1) **Certification process For Trainee**

After completion of service provider training, the participants will receive the training completion certificate.

## HEALTH FACILITY-BASED DIABETES EDUCATION TRAINING

Diabetes education for CHWs involves HA, AHW OR ANM. CHWs will have constant interaction within and across various units of health facilities. For example, clinician, medical officer or non- paramedics health care worker will be involved in proper diagnosis, clinical workup, treatment of diabetes including providing prescription and dispensing medicines; laboratory staff will be engaged in screening by doing blood test, nutritionist will be engaged in proper counseling on dietary modification; physiotherapist will be involved in prescribed exercise therapy and pharmacist for drug counseling. Depending on the staffing composition at a health facility, role delegation may vary slightly in different settings. However, the fundamental module in all settings is the CHWs being the nucleus of diabetes care being based on teamwork, shared responsibility, close coordination and sense of team belonging among potential health facility workers.

Engagement in a health-facility based approach not only has the potential to improve the quality of delivery of diabetes services but can also transform health service delivery by strengthening teamwork among health facility staff.

It is important to ensure that the diabetes training program conforms to the defined parameters, such as: exposure and dosage of lessons, and quality of the training content. Diabetes training should aim at enhancing knowledge and skills and build a culture of continuous learning among CHWs. The diabetes training program should be introduced in a health facility without interrupting the routine services.

## OBJECTIVES

| Objectives of Diabetes Training |
| --- |
| 1. Provide theoretical, practical and sustainable skills to participants about the diabetes 2. Prevention, early detection, treatment and management of major 4 diabetes according to the training manual 3. Increase capacity of regular follow up and availability of diabetes related medicine and equipment 4. Improve the knowledge and skills to the health workers related to the motivational and behaviors change counseling 5. Provide knowledge and skills to establishment and continuation of diabetes related prevention and control services at health facility and coordination at local level and community through community engagement and mobilization 6. Provide knowledge, skills and established the mechanism of recording, timely reporting of diabetes related data and information for necessary program monitoring and evaluation 7. Specially the decrease the intake of tobacco and smoking and alcohol, promote behavior to take healthy food and regular physical exercise |

**SCHEDULE FOR TRAINING FOR TRAINEES (3 WORKING DAYS)**

Schedule is developed for Diabetes Training for CHWs providing diabetes services.

| Time | Duration (Hour/Minute) |  | Activities/ Content |
| --- | --- | --- | --- |
| Day 1 |  |  |  |
| 9:00-9:05 | 5 mins |  | Welcome participants |
| 9:05-9:25 | 20 mins |  | Introduction of the trainers Introduction of the participants |

| 9:25-9:35 | 10 mins |  | Identify expectations of participants |
| --- | --- | --- | --- |
| 9:35-9:45 | 10 mins |  | Provide overview of the course and importance of TOT Share course goals and objectives |
| 9:45-9:50 | 5 mins |  | Review rules/ Group norms |
| 9:50-10:05 | 15 mins |  | Logistic arrangements |
| 10:05-10:25 | 20 mins |  | Pre-test Exam |
| 10:25-10:30 | 5 mins |  | short break |
|  |  |  |  |
| 10:30-10:40 | 10 mins |  | Tea break |
| Module1: Introduction of Diabetes, Risk Factors, Symptoms and Investigations of DM, Hypoglycemia, Complication of DM, Treatment and management of diabetes mellitus | | | |
| 10:40-10:50 | 10 mins |  | Diabetes Mellitus(DM) definition |
| 10:50-11:00 | 10 mins |  | Epidemiology and burden of Diabetes Mellitus (DM) |
| 11:00-11:30 | 30 mins |  | Types of Diabetes Mellitus:  Type 1 Diabetes Mellitus (T1DM) Type 2 Diabetes Mellitus (T2DM) Gestational Diabetes |
| 11:30-11:50 | 20 mins |  | Risk factors of T1DM,T2DM and GDM |
| 11:50-12:00 | 10 mins |  | Symptoms of DM |

| 12:00-12:30 | 30 mins |  | Regular Blood Glucose Monitoring |
| --- | --- | --- | --- |
| 12:30-12:50 | 20 mins |  | Acute complications : list and describe  **Hypoglycemia,** DKA, HHNK |
| 12:50-1:35 | 45 mins |  | Healthy Lunch break |
| 1:35-2:25 | 50 mins |  | Chronic Complications of DM, Treatment and management |
| Module 2: Blood Pressure and Diabetes  ` | | | |
| 2:25-2:45 | 20 mins |  | Blood Pressure and Diabetes |
| Module 3: Medication | | | |
| 2:45-3:30 | 45 mins |  | Medications: (Oral medicine, Injection Insulin) |
| 3:30-3:40 | 10 mins |  | Review |
| Day 2 | | | |
| 9:00-9:30 | 30 mins |  | Agenda Warm up  Review of previous day Test |
| Module 4: Physical Activity | | | |
| 9:30-10:30 | 1 hours |  | Exercise regime |
| 10:30-10:35 | 5 mins |  | Short break |
|  |  |  |  |
| 10:35-10:45 | 10 mins |  | Tea break |

| Module 5: Depression, Stress and time management | | | |
| --- | --- | --- | --- |
| 10:45-11:45 | 1 hours |  | Depression, Stress and time management |
| Module 6: Dietary habits | | | |
| 11:45-12:45 | 1 hours |  | Dietary Modification |
| 12:45-1:30 | 45 mins |  | Healthy Lunch Break |
| Module 7: Behavior Change: Alcohol consumption, Smoking | | | |
| 1:30-2:00 | 30 mins |  | Alcohol Consumption, Harmful use of Alcohol |
| 2:00-2:30 | 30 mins |  | Smoking |
| 2:30-3:30 | 1 hours |  | Practical  Create a video on the respective education session |
| 3:30-3:40 | 10 mins |  | Review |
| Day 3 | | | |
| 9:00-9:30 | 30 mins |  | Agenda Warm up  Review of previous day Test |
| Module 8: Foot care | | | |
| 9:30-10:15 | 45 mins |  | Foot care |
| Module 9: Oral Health | | | |
| 10:15-10:45 | 30 mins |  | Oral health in DM |

| 10:45-10:10:50 | 5 mins |  | Short break |
| --- | --- | --- | --- |
| 10:50:11:00 | 10 mins |  | Tea break |
| Module 10: Sexual Health | | | |
| 11:00-11:30 | 30 mins |  | Sexual health in DM |
| Module 11: Travel tips and Sick Days | | | |
| 11:30-12:00 | 30 mins |  | Precaution during travelling |
| 12:00-12:20 | 20 mins |  | Precaution during sick days |
| Module 12: Social and Emotional Supports / Meet to the health Professional | | | |
| 12:20-12:50 | 30 mins |  | Social and emotional support |
| 12:50-1:35 | 45 mins |  | Healthy Lunch Break |
| 1:35-2:05 | 30 mins |  | Meet with health Professional |
| 2:05-2:45  2:45-3:05 | 40 mins  20mins |  | Review and video on respective education sessions  Post Test |
| 3:05:3:35 | 30 mins |  | Certificate distribution |
| 3:35-3:45 | 10 mins |  | Wrap up |

## HOW TO CONDUCT DIABETES TRAINING EFFECTIVELY? GUIDE TO TRAINERS

**HOW TO BE AN EFFECTIVE TRAINER:**

### Prepare for the training

Whether trainers have been invited to facilitate a training session or are the focal point of a training session, he/she can prepare and organize themselves in advance in a number of ways to avoid obstacles during the sessions. A checklist can help trainer make sure they have the necessary materials and resources ready and that the venues and facilities meet their expectations.

## KNOW THE TRAINING ARRANGEMENTS:

- Check the training timetable
- Make sure trainers know exactly what day and time is scheduled to facilitate the training session.
- Check the venue for training.
- Take all relevant documentation (letters from the organization, outline the training details, names of people coordinating the training, ensure participants availability, the names of any support or administration staff who may be available to help, and the names of other trainers who may be attending your training session.

## KNOW THE MATERIALS:

- Trainers must be familiar with the materials they are presenting. Read over the materials before the presentation. Be prepared to answer questions about it. A reference list would be handy so trainers can let participants know where they can find additional information on a specific subject.
- A session plan can help guide the length of question time and when to suggest that the group move on to the next subject. Gather training materials, in more than one format, e.g., PowerPoint presentations. Ensure availability of necessary equipment, logistics, materials etc.
- Ensure availability of Trainers guide, Trainee's Manual, exercise sheet etc.
- Ensure availability of necessary adequate stationary.

## KNOW THE ENVIRONMENT:

★ Arrive early at the training facility and find out the location of the training room.

★ Get orientation to the area.

★ Make sure the training room is appropriate.

★ It should be large enough for all participants and is feasible to conduct the training activities e.g., for forming small groups. If the room is not adequate, inform the facility administrator and see if another room is available. Trainees can always be redirected to the new room as they arrive.

★ Minimize distractions.

- - If the environment is noisy or there is a great deal of movement in the corridors, etc., close the doors before starting the presentation.
  - If the doors are closed, the ventilation and temperature inside the room must be regulated to ensure comfort.
  - If trainers are the first to arrive, it‘s better to arrange furniture to suit the needs of the training. This can save time later.
  - Be familiar with the location of light switches and controls for blinds, curtain strings, etc.

★ Safe learning environment (Physical setup, surroundings for participants and speaker both) should be created.

★ Comfortable areas (comfortable chairs, and seating arrangements) should be planned.

Communication should be respectful, open and friendly.

★ Learning outcomes should be clearly stated for each session.

★ Information should be organized and delivered that addresses different learning styles.

★ Participants should be encouraged to share experiences and take advantage of the knowledge, skills and attitudes that already exist in the group.

★ Disrespectful or discriminatory behavior should be avoided.

## KNOW THE EQUIPMENT:

- Determine in advance, all equipment is available for training. It will be impossible to present a PowerPoint session when the facility has only an overhead projector available.
- Make sure all equipment and instruments needed are available. Check this off on the checklist.
- Check each piece of equipment to make sure it is working correctly. Make sure that the overhead machine or slide projector is focused adequately for the display of presentation. The screen should also be visible to all participants in the training room.

## KNOW THE AUDIENCE:

If possible, try to obtain a list of the trainees for the training course in advance. The list should contain their positions and place of employment. This information is important for a number of reasons:

- Knowing the number of trainees attending allows trainers to plan activities and group work adequately.
- Knowing the professions of the trainees, will give trainers an idea of the trainees‘level of education.
- Knowing the trainees ‘place of employment helps determine the following:
- The field each participant is working in so the various examples or case studies can be made relevant to their experiences,
- How many of the participants come from a similar organization.
- Knowing the trainees‘ positions will give trainers an idea of the range of seniority among the group. This may be important in identifying junior trainees so that they can be encouraged to contribute to the training session to the same extent as senior trainees.
- Knowledge of the average trainee‘s level of education and degree of background
- Knowledge allows trainers to pitch the training content and materials at the correct level. The trainees should not find the training too difficult or not challenging enough.
- Knowing the audience also gives trainers an understanding of the social and cultural background of the trainees.

## PRESENTATION SKILLS:

The presentation skills are broken down into a series of “micro skills” to make them easier to learn.

### Getting attention

One of the functions of the introductory part of the session is to gain the attention of the trainees. The trainer can gain attention by:

- Explaining how the session is relevant to the trainees;
- Asking the trainees what their expectations are from the sessions;
- Providing a relaxed and an open learning environment;
- Using humor or an activity as an icebreaker;
- Using novelty, variety, or a surprise in the introduction;
- Using a case study or narrating a story, relevant to the situation of the trainees;
- Using interesting pictures or seek audiovisual help at the start of the session; and
- Use quizzes as a means of identifying gaps in knowledge.

### Maintaining interest

For adults to focus on learning, they need to remain interested throughout the session. The trainees must recognize the relevance of the session and be able to participate in the sessions therefore every session has to be presented in an interesting way. The trainer can help the trainees remain interested by:

- Personalizing the presentation–smiling, making eye contact, and addressing trainees by name when interacting;
- Keeping the subject relevant and emphasizing how the topic relates to their needs;
- Being enthusiastic;
- Making sure the pace is neither too fast nor too slow;
- Using a variety of presentation styles;
- Introducing a new activity or providing valid information,
- Encouraging the trainees to participate;
- Using stories as examples;
- Having brief physical activity or game breaks;
- Using humor; and
- Using appropriate and consistent non-verbal behaviour (discussed on the following page).

### Selecting appropriate presentation styles

Using more than one technique in each session will help capture and retain interest by targeting different trainees’ learning styles. The technique used will depend on the following:

- Trainer–knowledge of topic and group, skills, personal style;
- Content–whether the aim is to learn knowledge or skills or change attitudes;
- Trainees–number, abilities, needs, and experience; and
- Environment–location, room set-up, time of day, day of week.

### The following activities can be used with groups of different sizes:

| Types of Activity | Large group | Small group | Pairs | Individuals |
| --- | --- | --- | --- | --- |

| Lecture | √ | √ |  |  |
| --- | --- | --- | --- | --- |
| Group discussion | √ | √ |  |  |
| Question and answer | √ | √ | √ | √ |
| Case study | √ | √ | √ |  |
| Brainstorming | √ | √ | √ |  |
| Quiz | √ | √ | √ | √ |
| Game | √ | √ | √ |  |
| Hypothetical Situation | √ | √ |  |  |
| Past Experiences | √ | √ | √ | √ |
| Problem solving |  | √ | √ | √ |
| Role play |  | √ | √ | √ |
| Demonstration |  | √ | √ | √ |

Presentation skills should be used effectively by the speaker to communicate in 3 different ways.

1. Verbally (through the words)
2. Non verbally (through body language, facial expressions and movements)
3. Para verbally (through the way of delivering words)

A speaker should be aware of what he/she is communicating through verbal, nonverbal, Para verbal to make speech more effective.

### Use Non-verbal communication

While watching someone present different types of information, more is learnt from his or her nonverbal communication (body language) rather than from spoken words. Non-verbal communication includes a range of signals which convey a message to the audience beyond what the speaker ‘s words may be. It can also prove to be a powerful tool, reinforcing what the trainer is saying, or it can contradict the trainer ‘s message. Trainers should try to be aware of their nonverbal communication messages.

### Non-verbal communication includes:

- - **Voice:** The trainers should speak clearly and project their voice. Displaying loudness and a high/ low pitch helps sustain the trainee’s interest. The voice should be audible and speech should be clear. The trainers can adjust their voice to emphasize important points that the trainees need to learn.
  - **Tone, speed and clarity of speech**: They should be maintained. The tone should match the message the speaker is trying to communicate with. Speech should be delivered at a reasonable pace that will allow people to easily understand without feeling like the speaker is rushing or nervous. On the other hand, avoid speaking slowly so that participants become bored or impatient.
  - **Selection of words**: Too technical words should be avoided if the participant has limited experience in the subject. Words, ideas or explanations should be used that they can relate to.
  - **Facial expressions**: It affects the sound of voice. Voice with a smile tends to sound more friendly and warm than a voice with frown or seriousness. Having a friendly facial expression and voice will help to make the participants feel safe and comfortable.
  - **Dress**. Wear formal smart dress. Looking good may also give the trainers added confidence.
  - **Eye contact**. In order to make the trainees feel involved, the trainers need to make eye contact with them. It is helpful to make regular eye contact for 1-3 seconds with participants.
  - In a large group trainers should try and make eye contact with as many trainees as possible.
  - **Posture**: Depending on the size of the group, trainers may need to stand upright to help project their voice to the whole group. Even within a small group, posture is important. The trainers should attempt to look relaxed (i.e, not stiff) without slouching or looking too casual (clasping arms together or keeping hands in the pockets).
  - **Position**: Where trainers stand is also important. When using audiovisual devices such as a board or a screen, trainers should stand back from the board or screen or to the side so that the device can be seen: If trainers have to write on a board, they should finish doing that first and speak to the trainees facing them. The room should be set up to minimize barriers of any kind.
  - **Movement and gestures**: Trainers should move about the room from time to time but not too often, since this may distract the trainees. Trainers should also use gestures for emphasis or explanation, as they would do in conversation, but these should also not be distracting.

## WHAT TO DO IF TRAINER BECOME NERVOUS:

Many people can get nervous before and during a presentation. Practice can help settle the nerves, but even the most experienced trainers may feel nervous before training sessions. Here are some ideas to overcome nervousness and anxiety:

- - Be well rested. Have plenty of sleep the night before and allow enough time to get to the training venue early.
  - Be well prepared and familiar with the session plan, and check everything on the training preparation checklist (review Session Plans).
  - Practice to have the presentation before the training session.
  - Greet the trainees as they arrive. If some trainees have friendly faces, trainers may not feel as if they are presenting to strangers.
  - Be relaxed.Try standing up or sit straight and breathing deeply. Tense and then relax the muscles and even do some stretching.
  - Have positive thinking and acknowledge well for being well prepared. Be confident in knowing the subject and that everything will be all right.
  - Wear comfortable clothes. If the dress is constricted and is hindering in moving freely around the training room, it will be difficult to present confidently.
  - Have a glass of water handy to reduce a dry throat or nervous cough.
  - At the start of the session, during the introduction session, trainers should give a short summary of the experience in the field. This helps to establish credibility and serves as a reminder that the trainer is the right person to be conducting the training.

## PERSONAL STYLE

- - There is no “right” way to train. Some of the characteristics of personal style are:
  - Use of appropriate humor
  - Use of relevant anecdotes
  - Personal enthusiasm
  - Self-confidence
  - Ability to develop rapport with trainees
  - Knowledge of the subject

## SELECTING APPROPRIATE AUDIOVISUAL AIDS

When choosing audiovisual devices, make sure they are relevant, simple, and not distracting. Fancy PowerPoint presentations with many colors and sounds can distract the trainees from the content. The technology available at the training venue, as well as its reliability, is also an

important factor to be considered. If no computers are available, PowerPoint is not a viable option. The following are some general tips for using audiovisual equipment.

- - Do not stand in front of or obscure the screen.
  - Use a pointer.
  - Cover all information until the trainers are speaking about it. Otherwise, trainees will read the information rather than concentrate on what the trainers are saying.
  - Make sure all the trainees can see the audiovisual device.
  - Talk to the audience, not the board or screen.
  - Check that all slides or overheads are properly focused before starting.
  - If using slides or computer projection, check that the room is not too brightly lit. Get help from someone to help to adjust the lighting.
  - Use only one audiovisual device at a time.
  - Have a backup. For example, if using PowerPoint™ slides, also have overhead transparencies or flex or chart papers just in case the equipment does not work.
  - Keep the layout simple, with minimum detail.
  - Use colors that can be seen clearly (not red or green for text).

### Some tips for using specific audiovisual equipment are as follows:

Power Point:

- - Keep the slides simple.
  - Avoid placing too much text on one slide.
  - Use two slides if the text is longer.
  - Avoid using many different colors and sounds.
  - Make the text large enough so it can easily be read by the trainees.
  - Keep the bullet points.
  - Avoid paragraph writing.

### Whiteboard:

- - Write legibly.
  - Use the right type of pen.
  - Cover or keep blank when not in use.
  - Use more than one color–preferably blue or black, which can easily be read from a distance.
  - Finish writing and turn towards the audience before speaking.

### Flipchart:

- - Cover pages that are not being used. Alternate blank and written pages.
  - The unit of message should be planned to be written on the individual chart in a logical order.
  - Appropriate and required number of paper sheets should be available to write down the units of message. Each unit of message should be brief.
  - The pictures and letters should be bold enough to be seen clearly from a short distance. Pictures should be attractive and captions appealing. Colors can be helpful to make the picture and caption clear and attractive.
  - Too many colors may complicate and create confusion. Two or three different colors are appropriate. Black, blue, green and red are commonly used.
  - Title of the flip chart should be written down on the front side of the cover.

### Poster

- - A good poster should be an appropriate size, normally of about 60 cm x 60 cm. The picture and letter should be big enough to be seen clearly from a distance of about 5 meters.
  - Color poster is more natural, attractive and clear.
  - Normally posters should be displayed at eye level.
  - The message should be based on the need of target people.

### Pamphlet

- - A pamphlet should be as brief as possible. It should not exceed more than 4 pages.
  - It helps in propagating messages rapidly in mass scale through wide distribution. They are easy to carry with from place to place.
  - Participants can read them in their free time and understand the message well.

### Flash Card

A simple guide regarding the size of flash cards in relation to the size of the audience group, and the distance between the presenters and learners is given below.

| Size of Flash Card | No. of Audience | Distance |
| --- | --- | --- |
| 8 cm x 12 cm | 4 to 8 | 60 cm to 80 cm |
| 12 cm x 18 cm | 9 to 12 | 80 cm to 100 cm |
| 18 cm x 18 cm | 13 to 19 | 100 cm to 125 cm |
| 18 cm x 25 cm | 20 to 25 | 125 cm to 150 cm |

### Handouts:

- - Consider an appropriate time to hand these out. When distributed at the start of the presentation, the trainees may focus on reading the handouts and not listen to the presentation.
  - On the other hand, distributing handouts early can enable the trainees to follow the discussion without taking notes.

## TRAINING METHODOLOGY:

The training has been designed to be fully interactive on the part of the participants, and to enable them to learn at an optimum level. In order to do this, various methodologies can be used including:

1. Short lectures/ presentations
2. Group discussions/work
3. Role plays
4. Demonstrations
5. Brainstorming
6. Case studies and reports
7. Games
8. Videos

### Short lectures/presentations:

Short lectures and presentations are used to provide basic information on a particular topic. Visual aids illustrating major points are also used.

- - The subjective and objective of the lecture should be related to the needs and interest of the target audience.
  - The speaker should get thorough and up to date knowledge of the content.
  - The language should be correct, simple, clear and understandable.
  - Monotonous voices should be avoided. Pitch of the voice should be changed appropriately.
  - A long speech cannot gain constant interest of the audience, and it adds to monotony, boredom etc. A single lecture should be reasonably brief and maybe about 25 minutes.
  - The speaker should be sincere, pleased and properly dressed up.
  - Trainers/facilitators are encouraged to facilitate full participation from their audience during mini-lecture sessions by:

★ Asking questions and encouraging participants to ask questions

★ Designating group exercises and presentations

★ Brainstorming among the participants

★ Problem-solving case histories

- - Following group work and presentations from the participants, the facilitator, with the help of the participants, lists the major points and summarizes the topic using these presentations.

### Group discussions/work:

Depending on the number of trainees, it is recommended that groups of four to six people be formed.

Each group should be given a task to carry out. This helps participants become actively involved in problem-solving and more comfortable with sharing their experiences. It also makes for an interesting and stimulating session, as each member will have to be prepared for a group presentation and be ready to answer questions from other participants. Additionally, this approach allows for the development of personal relationships. Such group work also helps the facilitator evaluate the trainees’ existing knowledge on the topic, and their capacity for absorbing the material being taught. This is also useful in designing future training sessions.

- - Before the start of the discussion, background information about the topic should be provided.
  - Sufficient time should be spent preparing the process and steps of discussion.
  - Different aspects of the topic and the parameters should be selected for the focused discussion.
  - Sufficient time should be allotted to discuss all the issues. At the same time, participants should know the time limit to reach a conclusion.

### Role-play

- - During a role-play session, the facilitator explains the objectives of the topic being covered (i.e. effective communication or history-taking).
  - Participants are asked to form groups and choose individual roles.
  - Appropriate topic for role playing should be determined. It must be based on the learner's need and interest.
  - Appropriate time, situation and place should also be determined. Arrangement of seating, lighting etc. should be made properly. Stage should be set appropriately so that the audience can watch the play well.
  - Necessary materials should be collected and managed to be used in role playing.
  - The facilitator should explain the scenario to the group and instruct each individual to play their role as convincingly as possible.
  - Participants forming part of the audience are instructed to observe the scenarios carefully and to provide feedback or comment on what they see once it is completed.
  - The facilitator should be prepared to help or guide the role-play session when necessary, and to encourage observers to comment on the positive and negative aspects of what they witnessed.
  - The role play should be discussed at the end and evaluated for its success and effectiveness through the interaction of the audience. The characters, the audience and the health educators can get involved in the discussion and pass comments.

### Demonstration

- - The topic, objectives and content to be dealt with in the demonstration should be identified.
  - Proper place and seats should be arranged to ensure a clear view by all learners.
  - Materials should be set up to be used in the demonstration in proper order of presentation.
  - Proper introduction should be given by the trainer if the participants are of a new group.
  - The topic and purpose of the demonstration should be clearly explained.
  - Demonstrations should be conducted step by step as planned. Arrangement should be made in such a way that each learner can see the demonstration well.
  - Participants are asked to carefully observe so that skills demonstrated can be included in their practice.
  - Eye contact should be maintained and each step should be checked by asking questions if it is understood well before going to the next steps.
  - Equipment and materials that are used in the demonstration should be carefully dismantled and replaced properly. They can be used in future demonstrations.
  - After the demonstration, the facilitator interacts with the participants and asks them to provide feedback. The facilitator then provides his own comments, answers any queries the participants might have, and later emphasizes the significant aspects covered in the session.

### Brainstorming

- - Brain-storming sessions are used to extract knowledge from participants on specific topics. These help participants to engage, allowing them to become attentive and alert which is conducive to effective learning.
  - During the brainstorming sessions the facilitator raises several questions on the topic being studied.
  - Participants are given a few moments to think about a given topic.
  - Ask participants to respond to them either individually or as a group.
  - The answers are to be written down according to their level of importance on a whiteboard or flipchart, and read aloud.
  - There should be no criticism and wild ideas should be encouraged and recorded without evaluation.
  - There is a need for equal participants of members.
  - It can be unfocussed, should know how to control discussion and facilitate issues.
  - The facilitator then provides his/her own comments, highlighting the positive aspects of the outcome, and does a follow-up presentation.

### Case Study

- - Case studies are designed to help participants acquire the management and confidence necessary for dealing with patients experiencing various conditions.
  - The cases presented are real or imaginary, characteristic of problems related to diabetes mellitus.
  - Case should be brief, well written, reflect real issues and open to a number of conflicting responses.
  - Should work in group to prepare a written report and/or a formal presentation of the case.
  - The participants are guided on how to locate the characteristics through a number of sequential steps.
  - Case studies can be used to introduce a particular training session or topics, assess participants‘ knowledge of the disease, or used as a follow-up exercise after the completion of a specific session.
  - The facilitator provides a case history and asks participants about the correct approach for that condition.
  - Conclusion of the discussion should be made clear to every member of the learning groups.

### Symposium

- - The ideas should be set including the goal and content of teaching, and identify the speakers.
  - Speakers should be informed ahead of time to get prepared on the topic. The content and purpose of presentation, and also about the nature of the audience should be briefly explained to the speaker.
  - Speakers and audience should be invited to meet at a particular time and place.
  - Physical arrangement of seating, lighting, ventilation etc. should be made.

### Workshop

- - Need and purpose of the workshop should be determined.
  - Workshop schedule should be developed.
  - Budget should be prepared and the suitable place should be fixed.
  - The proposed participants should be invited stating the purpose, time and the place of the workshop.
  - Necessary equipment, materials and teaching aids should be arranged.
  - Resource person or consultants should be invited to help organize as well as conduct the workshop well and achieve workshop objectives.

### Exhibition

- - The target group should be decided and objectives should be determined based on their needs.
  - Necessary materials and exhibits should be prepared. Necessary budget and other resources can be tapped from different sources.
  - Exhibition should be planned in a logical and sequential way at eye level.
  - Exhibition should carry appropriate captions. The captions and other visual messages should be brief, simple and clear.
  - Proper provision of physical facilities of lighting, tables, chairs, racks etc. should be maintained.
  - The members of the target group should be given the opportunity to participate in the organization of the exhibition. It is very good from the education point of view.

**Note:** Short games can be used to energize the participants during the sessions wherever appropriate.

## MANAGING COMMON DIFFICULTIES IN TRAINING

Even the most experienced trainers can face difficulties while presenting or facilitating a session with a group. It is important to be aware of common problems and to understand ways to address them.

No one is a perfect trainer; we all have shortcomings which we constantly need to be aware of when managing a session. Below are common problems and practical responses to get the session back on track.

### Mixed group expertise and experience (high to low)

The trainees may have a wide range of knowledge and experience. Some of the following strategies can be effective in meeting this challenge:

- - If high expertise trainer is there then use as more as examples rather than content as per participants knowledge status and their capacity to learn, e.g., to provide examples based on their experience.
  - Split the trainees into different groups on the basis of ability, knowledge, or experience.

### The trainee who doesn’t want to be there Early in the session

The trainer will become aware that one or more persons would rather not be at the training session. They may be indicated by being unwilling to participate in activities, talking to others, or just generally showing disinterest. In response, the trainer can:

- - Ask the persons how they feel about being present at the training.
  - Offer them the option of leaving the training: “It is OK for me if you don‘t want to stay.” usually they will choose to stay.
  - Ask them what can be done to make the session relevant to their needs. You could perhaps clarify their objectives in attending the training and suggest how the training can meet their needs.

### Late arrivals

Enforcing punctuality among trainees can be a challenge. Those who arrive late can delay the start of the session or disrupt training that has already begun. Other trainees should not be penalized for the late arrival of others.

- - At the onset of training it is important to stress the necessity of arriving on time to allow the training to start at the designated hour.
  - Tell the trainees that the training session will begin at the designated time and will not wait for people to arrive.
  - Set group rules. Most groups usually agree that punctuality is important. Peer group pressure can be very effective in encouraging trainees to be punctual.
  - See to it that all trainees are aware of the timetable. Ask them if they are happy with the current timetable and if there is any reason that they cannot arrive on time.
  - Stress the importance of punctuality in any promotion or invitation letters for the course.

### Non-attendance

Attendance of the trainees for all sessions and their entire duration is important. People who leave early or skip sessions can slow down the progress of the group, as they will need time to catch up. If they| have been assigned to a particular group, the rest of their team is at a disadvantage. To help ensure full attendance at the training:

- - At the start of the training, inform the trainees that those who do not attend the whole course will not receive certificates (unless they have a valid reason for being absent and miss only a small part of the training).
  - A trainee who misses any segment should be briefed on his or her return about the portions missed.
  - If a trainee cannot complete a course due to an emergency, negotiate with his or her trainer to complete the missed segments at a future course and obtain a certificate at that time.

### Lack of time

Trainers often run out of time. It is easy to underestimate the time needed to teach a certain subject. This holds true especially with regard to group activities which generally take longer than expected. Time management may also be a problem if the trainer is teaching a particular session or conducting a training program for the first time. Use the following strategies to keep on time:

- - Keep an eye on the time. Check it regularly but discreetly. Use of a session plan to allocate the time needed for each topic.
  - Skip topics and refer to the reading list if there are subjects that cannot be covered during the time available. Avoid skipping planned activities as these are an important part of reinforcing the learning process.
  - Acknowledge the problem and negotiate with the trainees for an extension of time.
  - Provide an overview of the remaining material and ask the trainees what they consider important and relevant to their work.
  - Offer to forward to the trainees a summary of the remaining material.

### Equipment failure

Virtually trainers may face equipment failure at some point in their career. The more sophisticated the technology, the more likely it is to malfunction or cause difficulties. Preparation is the best strategy for avoiding equipment failure or overcoming it.

- - Check the equipment. Make sure it is working, although sometimes equipment failures are unavoidable. Arrive early and familiarize with the equipment especially if trainers have not used it before. Check the source of power.
  - Apologize and remain calm. Tell a joke and move on.
  - Write key points from manuals on newsprint or on a whiteboard.
  - Know the subject so trainers can present without equipment. A good trainer who is well prepared should be able to present without the aid of sophisticated technology.

### When trainees do not respond to calls for feedback or questions after a focal activity

Questions can be thrown to the participants:

- - **Open and closed questions**: -Open questions are much more likely to get a response. The differences between closed and open questions are illustrated below.
  - **Closed**: ―”Any questions? Any point’s people want to raise?”
  - **Open**: ―”What are some of the key points raised by the session/video?”

### For dominating trainees

- - **Be respectful and courteous.** Trainees are unlikely to respond if trainers are angry or aggressive. Be assertive and confident in the trainer's manner.
  - **Verbal responses**. Trainers can try a range of verbal strategies. For example, “Thank you very much. I would now like to hear what (use name) has to say on this topic.” Do not say “Why don‘t we come back to this later?” If you do not intend to return to the topic.
  - **Non-verbal responses**. Trainers can orient their body away from the dominating trainee so trainers can disengage from eye contact and body language discourages him or her from continuing to speak. Combine this with a verbal response such as inviting another trainee to contribute.

### Unresponsive trainees

Some groups are naturally talkative and easy to work with. Others are unresponsive and may require trainers to call on additional techniques to engage them.

Trainers can try the following:

- - **Use silence to pressure the group**. Ask a question that trainers know someone in the group can answer and wait for an answer. Trainer can remain silent and better not answer the question. Eventually (in most cases) someone will respond.
  - **Identify** one or two people in the group whom the trainer can ask to say something.
  - **Be controversial or challenging**. Used carefully, this technique can get a group going. There could be controversial issues within the training session, so finding something that challenges the group at some level should not be too difficult.
  - **Ask for feedback**. Say: “I sense that there is not a lot of interest in this subject” or “I sense that you feel this subject is not relevant to you.”
  - **Introduce an activity,** something to energize the trainees and get them to respond either as a whole or in small groups.

### Sleeping or inattentive trainees

- - **Walk near the person,** while talking to the group. Do not single the person out by looking directly at him or her. Stand next to the person for a while without necessarily looking at or drawing any other attention to him or her.
  - **Throw a question at the inattentive person**, but remember to allow him or her to save face. Ask a question that the person is likely to know the answer to, or provide a quick summary of the current issue and then ask the question. DO NOT say, “Why you were asleep…” rather say, “Let me explain what we are up to.”

### The argumentative trainee

Some trainees may be argumentative. They may be genuinely upset or disturbed by something and choose to demonstrate this by arguing with the presenter or other members of the group.

- - **Don’t get hooked into the power struggle**. It is not the duty of a trainer to win the argument, even though the trainer may strongly disagree with the person‘s opinion. The more trainers assert their opinion, the more likely it is that the person will stop listening to the trainers.
  - **Don’t use personal attacks**. In challenging the argumentative trainee, do not use personal attacks. These tend to put people on the defensive and undermine credibility of the trainer as a facilitator.
  - **Use assertive communication**: “I can see how you would think that. However,…”; “Some people feel that…”; “There is a range of opinions on this subject…”.
  - **Redirect discussion to other trainees**. Ask if anyone else in the group has a different opinion.
  - **Use direct and calm but assertive body language**.

## EVALUATING THE TRAINING SESSION/ COURSE EVALUATION

Many stakeholders are involved while conducting the training sessions including the trainer, the trainees, the training institution, and the organization purchasing the training. Different stakeholders may have different expectations of the training and anticipate different outcomes. It is important to speak with different stakeholders to understand what they need to know about the training.

### What are the benefits of evaluating the training?

Evaluating specific aspects of training can benefit all stakeholders. The possible benefits may include the following:

### For trainers:

- - Information regarding ways to improve the training (contents, process, tools);
  - Information about possible improvements in training process, style and skills.

### For trainees:

- - Assessment of whether they have achieved their learning goals;
  - Consideration of how the knowledge and skills learned can be applied to their work;
  - Decisions about whether training has been a worthwhile investment of time, effort, and money.
  - Theory evaluation: There will be a pre-test before starting each module and post-test after completing the module. There will be a final theory exam (Multiple Choice Questions) at the end of the training.
  - Practical evaluation: Trainee should conduct at-least one education session/ counseling during the training period.

### For Management offices and sponsors:

- - Information about the extent to which the training was worth the time and money they invested in it.
  - Information about staff that are capable, including their limitations and readiness for new responsibilities.

### What does evaluation measure? Goals.

**Inputs.** Evaluation can give us information about:

Training tools:

- - Was the course content targeted at the appropriate level for the trainees?
  - Were the handouts easy to understand?
  - Was the appropriate audiovisual equipment used?
  - Did the audiovisual device work?

Training environment: Were the training facilities (e.g., room size, ventilation, temperature,

refreshments, and audibility) adequate?

**Processes**. Evaluations can tell about the quality of the training, including the following: Training framework:

- - Was the training too long or too short?
  - Were there enough breaks?
  - Were the sessions in logical sequence?

Training techniques:

- - Was a variety of techniques (e.g., group work, role plays, games, exercises, didactic

teaching) used?

- - Which techniques worked best?

Trainer’s style:

- - Did the trainer have good teaching skills (e.g., maintained the interest of the group, used a variety of teaching techniques, facilitated discussions, and created a supportive environment for trainees)?
  - Was the trainer friendly, personable, approachable?
  - Did the trainer know the materials (e.g., could he or she answer questions about the

materials confidently)?

**Outputs.** Evaluating outputs can tell us about the immediate benefits of training, including the following:

Change in trainee knowledge: Trainers need to be sure that trainees have understood the course content.

Trainee satisfaction:

- - Did the course meet the trainees’ expectations?
  - What did the trainees like about the course and what didn‘t they like?

### Methods and tools used to evaluate training.

A number of methods and tools can be used to evaluate training. These include evaluation by the following:

### Trainer:

- - A checklist for pre-training evaluation to assess readiness for training, e.g., to check that the necessary equipment, materials, and tools have been prepared and are ready (quantitative);

### Trainees:

- - A training evaluation form, mostly for assessing training processes (quantitative and qualitative measures);
  - Pre- and post-course knowledge tests for trainees;
  - Assignments or “homework”;
  - Discussion questions at the end of each session to assess level of knowledge and understanding;
  - Problem solving using a case study and information discussed previously; and
  - Skill testing through role-play.

# DAY 1

## WELCOME AND INTRODUCTION

### Arrival and welcome

- - Welcome by representatives of the organizations conducting an orientation program.
  - Time allocation: 10 mins

### Introduction

- - Time allocation: 20 mins

1. Introduction of the trainers: Trainer will introduce herself/ himself including name, designation, work experience and role for the training.
2. Introduction of the participants:

Ask the participants to introduce themselves by keeping adjectives in front of their name.

## COURSE INTRODUCTION

### Identify expectations of participants

- - Time allocation: 20 mins
  - Ask participants to share expectations of the course on metacards. Record their response on the flip chart or attach their metacards on the flip cart and share in the group.

### Provide overview of the course

- - Time allocation: 10 minutes
  - Review the course syllables and schedule.

### Share course goals and objectives

- - Time allocation: 5 minutes
  - Discuss goals of the course and participants learning objectives.
  - Review table of contents of the reference manual and participant handbook.

### Review rules/ Group norms

- - Time allocation: 5 minutes
  - Trainer will convey the general rules and norms of the training to the trainees. They include:
  - All trainees should be punctual for both theoretical and practical classes.
  - All trainees must maintain good interpersonal relationships and proper communication with all the members of the training, hospital staff, patients and patient family members.
  - All trainees should keep their mobile in silence or vibration mode during class hours. Attendance is a must for all the attendee. 90% attendance is a must to get the certificate.
  - Prior information should be given to the trainer in case of absence.
  - Given assignments should be done by the trainee.
  - Respect each other.

Day activities can be planned in the following way:

| Date | Agenda | Review | Summary | Officer of the day |
| --- | --- | --- | --- | --- |
|  |  |  |  |  |
|  |  |  |  |  |
|  |  |  |  |  |

### Logistic arrangement

- - Time allocation: 15 minutes
  - Arrange all the required logistic materials like

a) Furniture b) Computer

1. Projector d) Pointer

e) Multiplug f) Stationeries:

White board; Markers; Dusters; Notice boards; Thumb pins; Pen Pencil; Eraser; Ruler; Sharper; Books; Manuals; Exercise copies; A4 size copies; Chart papers; Sign pens; Colours; Metacards; Cello tape; Index papers; Files.

1. Audio Video Aids:

Flex consisting picture of pancreas; Risk factors of diabetes, symptoms of diabetes Chronic complication of diabetes; Hypoglycemia and its management; Diabetes plate model; Flex of exercise; Photo gallery of foot care; Flip flex of foot care.

1. Anthropometric measurement scales:
   - Blood pressure instrument
   - Inch tape
   - Stadiometer (Potable)
2. Miscellaneous:
   - Water dispenser
   - Water bottle
   - Plastic glasses
   - Tissue paper

Distribute review and discuss materials used in the course.

### Pre-Test

Assess participants pre-course knowledge

- - Time allocation: 20 mins
  - Distribute Pre-test questionnaires (attached in the annex) and instruct participants to answer questions in the given time.

1. **Short break** Time allocation: 5 mins

### Tea break

- - Time allocation: 10 mins
  - Trainer will reveal the agenda for the day. The agenda includes coverage of the introduction of Diabetes Mellitus (DM).

## MODULE 1: INTRODUCTION OF DIABETES, RISK FACTORS, SYMPTOMS AND INVESTIGATIONS OF DM, HYPOGLYCEMIA, COMPLICATION OF DM, TREATMENT AND MANAGEMENT OF DIABETES MELLITUS

Instruction to the trainer: Trainer will post a chart consisting learning objectives of Module 1, “Risk factors, symptoms and investigations of DM”, Complications and management of DM . The learning objectives are as follows:

### Learning objectives of the module:

- - To define Diabetes Mellitus (DM)
  - To identify current epidemiology and burden of DM
  - To explain normal physiology of glucose metabolism and pathophysiology of diabetes mellitus To classify DM and explain their pathophysiology
  - To enlist the risk factors of DM
  - To enlist the symptoms of DM
  - To explain investigations related to DM (Tests and check-ups)
  - To elaborate acute complications of DM, Hypoglycemia
  - To explain chronic complications of DM
  - To explain diabetes management process as a treatment

### Contents of the module:

- - Definition Diabetes Mellitus (DM)
  - Current epidemiology and burden of DM
  - Normal physiology of glucose metabolism and pathophysiology of diabetes mellitus
  - Classification DM and explain their pathophysiology
  - Risk factors of DM
  - Symptoms of DM
  - Investigations related to DM (Tests and check-ups)
  - Acute complications of DM
  - Chronic complications of DM
  - Various psychological and behavioral strategies for self-management

### Time allocation for the module:

- - Total time: 1 hour 50 mins

# Module 1.1 Definition of Diabetes Mellitus (DM)

Time allocation: 10 mins

Instruction to the trainer: Trainer will ask the trainees to share their experience related to diabetes mellitus. That may include knowledge on diabetes, work experience related to diabetes, any significant diabetes cases encountered during the work, any family history, any personal history among others. Opportunities will be given to all the trainees if they want to share their experience briefly regarding DM.

Trainer will also ask the definition of diabetes mellitus. The floor will be opened for voluntary participation. Only three answers will be included to explore the definition of diabetes. Trainer will use power point and flex comprising anatomical picture of pancreas.

Additional note to trainer:

Insulin resistance is caused by:

1. Increased action of lipoprotein lipase and decreased lipogenesis.
2. Leptin resistance (Leptin causes increased appetite, increases glucose use for energy by causing insulin sensitivity, but in Type 2 Diabetes Mellitus (T2DM), there is leptin resistance).
3. There is increased oxidative phosphorylation leading to increased triglyceride accumulation in muscles.
4. Interaction between genetic pre-deposition, diet and intestinal microbiota.

### Module 1.2 Epidemiology and burden of DM

Time allocation: 10 mins

Instruction to the trainer: Trainer will ask trainee if they know prevalence of DM in the world and in Nepal. The participation will be voluntary. Trainer will use a lecture method to discuss epidemiology of diabetes mellitus using powerpoint and metacard.

### Mortality due to diabetes and Economic Burden

Instruction to the trainer: Trainer will ask if trainees know about how much economic burden created due to diabetes and mortality caused by diabetes in the global context and in the context of Nepal. The participation will be voluntary. Trainer will use powerpoint to discuss the economic burden created by DM and mortality due to diabetes using lecture methods.

### Module 1.3 Classification of diabetes mellitus and their pathophysiology

Time allocation: 30 mins

Instruction to the trainer: Trainer will do brain storming to the trainees if they know different types of diabetes mellitus. Trainer can probe participants to let the trainee speak out to tell different types of diabetes mellitus they know or they have encountered. Trainer will write the names of different types of diabetes mellitus on the white board based on the answers of the trainee. Trainer will appreciate trainee if they correctly name the types of diabetes mellitus. Trainer will correct the trainee if they do not give the right answer. Trainer will also ask trainees if they know anything about pathophysiology of diabetes and its types. Trainer will use a lecture method using power-

point to explain types of DM and their pathophysiology. Trainer will also use case study to let the participants have clear ideas about types of DM.

Note to the trainer: The manual consists of detailed information on T2DM and brief information about T1DM and Gestational Diabetes Mellitus (GDM). Other types of diabetes are not covered in detail in this manual.

### Module 1.4 : Risk factors of T1DM, T2DM and GDM.

Time allocation: 20 minutes

Instruction to the trainer: Trainer will divide trainees in three groups. Then the trainer will ask three groups to write down risk factors for Type 1 Diabetes Mellitus (T1DM), Type 2 Diabetes Mellitus (T2DM) and Gestational Diabetes Mellitus (GDM in index paper. 5 minutes will be given for this. The answered index papers will be posted on the notice board and let the representative of the group present their respective topics. Maximum 5 minutes will be given to each group for this.

### Module 1.5 Symptoms of DM

Time allocation: 10 mins

Instruction of the trainer: Trainer will ask if trainees know about different symptoms of diabetes. Trainees can simply raise their hand turn by turn and answer the question. Trainer will write the answers in white board. Then the trainer will summarize the symptoms by using powerpoint.

### Group assignment

Instruction to the trainer: Trainer will divide three random groups or more if the number of trainees is more. Then the trainer will provide three cases of diabetes. Trainer will ask the group to read the case, evaluate the case, diagnose the case, explain the symptoms and investigation as well as recommend. Trainer will ask the group present the case along with the answers related to the case. 5 mins will be allocated for each group to present their case. Trainer will give feedback to the presenters at the end of each presentation.

The three cases are as follows:

**Case 1**: A young male aged 18 years having symptoms of excess thirst, excess urination, excess food intake and decrease in weight in a few weeks, came for check-up. The blood test showed high random blood sugar (350 mg/dl) and HbA1c (14%). He was then diagnosed with diabetes. To rule out the type of diabetes, c-peptide was sent, which showed reduced c-peptide.

**Answer:** Case: T1DM

Symptoms: Excess thirst, excess urination, excess food intake and decrease in weight Investigations: Random blood sugar ,HbA1c, c-peptide

**Case 2**: A middle aged obese woman had symptoms of increased thirst and urination along with itching of vagina. She came for a hospital checkup. In history taking, her father had diabetes. Her weight was reduced by 7 kg within a month, though she was obese during presentation. She had her blood test. Random blood sugar was 600 mg/dl and HbA1c was 11%.

**Answer:** Case: T2DM

Symptoms: Excess thirst, excess urination, excess food intake and decrease in weight Investigations: Random blood sugar ,HbA1c.

**Case 3**: A happy pregnant lady at her 26 week of gestation came for her regular checkup in her Ante-natal care unit in the hospital. She undergoes regular checkup of Oral Challenge Test (OGT). For this, she is given 50 mg anhydrous glucose. After an hour, she will have her blood sugar test, which was higher than 140 mg/dl. Then she will be recommended for hospital admission to have a second test called Oral Glucose Tolerance Test (OGTT) in the next day in a fasting phase of 8 hours. So she gets admitted. She will give her fasting blood sugar in the morning. Then she is given 100 mg of anhydrous glucose as per the protocol of Dhulikhel Hospital. However, for this test, 75 gram is given as per the need. Then she gave a subsequent 3 blood sample in 1 hour, 2 hour and 3 hour to determine blood sugar. Out of these tests, two of her blood tests will show above normal.

**Answer:** Case: GDM Symptoms: Excess thirst, excess urination, excess food intake and decrease in weight Investigations: Random blood sugar ,HbA1c

### Module 1.6 (Regular Blood Glucose Monitoring)

Time allocation: 30 mins

Instruction to the trainer: The trainer will explain the need for regular blood glucose monitoring. How often there is a need for regular blood checkup will be also elaborate.

### Equipment used in Diabetes Care

Instruction to the trainee: Trainer will now move on to another titled “Equipment used diabetes care”. For this, trainer will brainstorm to the trainee if they can name different equipment used in diabetes care. Trainer will give time to the trainee and will write the name of the equipment mentioned by trainee on the white board. Trainer will begin the lecture using powerpoint and different available gadgets.

**Note for the trainer:**

Glucometer may be of different companies such as One Touch, Accu Check, Gluco Card, Oncall Plus, Omni test (B-Braun), Clever check and so on. Glucometer are of two types, Coding Chips (Strips) and No Coding Chips (Strips). Among these two No coding chips glucometer is the most effective one for all diabetes patients.

Coding Chips glucometer required some code number which is fixed one so that other chips or strips cannot be used. E.g. Code P20 strips only works in one touch glucometer.

**Additional note for the trainer:**

Mechanism of SMBG: It includes technology that determines the blood glucose level using SMBG. Glucometer is a portable electronic blood glucose meter that is used for monitoring glucose level. Glucometer measures blood glucose by using color reflectance or Sensor technology

Reflectance photometry: A detector captures the reflected light and converts it to electronic signals which translated to its corresponding glucose concentration. The lower the glucose, lighter the color and vice-versa.

Biosensor Technology: A biosensor is an electronic device that quantifies the number of electrons generated by oxidation of glucose i.e. it measures the electric current. Using enzyme catalyst, glucose is oxidized with a mediator to generate electrons which subsequently detect blood glucose level.

A detector converts the resulting current to an electric signal and translates that signals to its corresponding glucose concentration. A number of electrons captured by the mediator are directly proportional to the amount of glucose present in the sample.

### Practical work

Instruction to the trainer: Trainer will arrange all the materials required for the glucometer test to demonstrate the use of glucometer for testing blood glucose levels . The required materials are:

- Glucometer
- Lancet
- Battery
- Cotton
- Spirit
- Safety box

### Module 1.7: Acute Complications: Hypoglycemia, DKA, HHNK

Time allocation: 20 minutes

Instruction to the trainer: Before moving to the proper topic, the trainer will verbalize one case, which comprises a diabetes patient who is under hypoglycemic agents and takes fasting for a day

due to puja at home. In the afternoon, the patient was found lying unconscious on the floor. Now, the trainer will ask trainees if they could recognize the situation of the patient.

Instruction to the trainer: If trainees answer the situation as hypoglycemic state, trainer will appreciate trainees for the right answer. Then the trainer will start the content of “hypoglycemia” properly using flip charts, real materials like glucometer, strip, cotton, spirit, chocolates, glucose powder among others.

Content includes Introduction of Hypoglycemia, Causes of Hypoglycemia, Symptoms of Hypoglycemia .

**Additional note to the trainer:**

Clinical classification of hypoglycemia: Severe hypoglycemia: It is a condition requiring assistance of another person for the management of hypoglycemia.

Documented symptomatic hypoglycemia: It is a condition when typical symptoms of hypoglycemia are accompanied by a measured plasma glucose concentration ≤ 70 mg/dL (3.9 mmol/L).

Asymptomatic hypoglycemia: It is a condition accompanied by typical symptoms of

hypoglycemia but with a measured plasma glucose concentration ≤ 70 mg/dl (3.9 mmol/L).

Probable symptomatic hypoglycemia: It is a condition in which symptoms of typical hypoglycemia are not accompanied by a plasma glucose determination but that was presumably caused by a plasma glucose concentration ≤ 70 mg/dl (3.9 mmol/L).

Pseudo hypoglycemia: It is a condition when a person experiences typical symptoms of

hypoglycemia but with a measured plasma glucose concentration ≥ 70 mg/dL (3.9 mmol/L).

- Relative hypoglycemia: It is a condition when a diabetes patient reports any typical symptoms of hypoglycemia but with measured plasma glucose concentration ≤ 70 mg/dl (3.9 mmol/L). These are the symptoms patients report due to decline in glycaemia based on the relative higher glycaemia during their chronically poor glycemic control.

## HEALTHY LUNCH BREAK

Time allocation: 45 minutes

### Module 1.8 Complication of diabetes mellitus, it's treatment and managements

Time allocation: 30 minutes

Instruction to the trainer: Trainer will ask trainees what are the acute and chronic complications, its treatment and management of DM. Trainer will give time to the trainees to think. Trainer will let the trainees answer on a voluntary basis. If trainees answer diabetic retinopathy, diabetic neuropathy, diabetic nephropathy, ischaemic heart disease, stroke, diabetes foot among others; trainers will appreciate trainees who answered right answers.

## MODULE 2. BLOOD PRESSURE AND DIABETES

Instruction to the trainer: Trainer will continue talking on blood pressure and its link to diabetes

### Learning objectives of the module:

- To describe the blood pressure in DM
- To explain good management of blood pressure in diabetes.

### Content of module:

- Blood Pressure and Diabetes
- Causes of blood pressure.
- On what often blood pressure should be checked.

**Time allocation for the module**: Total time: 20 mins

## MODULE 3. MEDICATIONS FOR T2DM

Instruction to the trainer: Trainer will begin the new module of the day as “Medical treatment for diabetes”. Trainer will put the topic on the notice board.

### Time allocation for the module:

Total time: 45 mins

### Learning objectives of OHA:

- To explain different categories of diabetes medicines
- To explain different diabetes medicines under different category

### Learning objectives of injectable hypoglycemic agents:

- To elaborate types of insulin and insulin regime
- To demonstrate and re-demonstrate insulin therapy

### Content of module:

- ORAL HYPOGLYCEMIC AGENTS (OHA)
- INJECTABLE HYPOGLYCEMIC AGENTS

## ORAL HYPOGLYCEMIC AGENTS (OHA)

Instruction to the trainer: Trainer will ask trainees if they know the names of different oral medicines used for diabetes. Trainer will give time to the trainees to answer. Trainer will give the opportunity to all the trainees sequentially to name one OHA. Trainer will also ask them to write the name of OHA in the metacard. Those metacards will be attached on the notice board. This will be useful during the lecture of OHA.

## PRACTICAL WORKS

Make a formulary for different drugs of Oral Hypoglycemic Agents. The names of OHAs are:

1. Metformin 11. Sitagliptin
2. Glibenclamide 12. Linagliptin
3. Glipizide 13. Vildagliptin
4. Gliclazide 14. Saxagliptin
5. Glimepiride 15. Canagliflozin
6. Repaglinide 16. Dopagliflozin
7. Pioglitazone 17. Empagliflozin
8. Acarbose
9. Voglibose

Note: Formulary should be in the following format:

- - Generic name of the drug
  - Pharmacological category
  - Similar drugs (any 3)
  - Brand names (any 2)
  - Mechanism of action
  - Indications
  - Adverse effects
  - Contraindications/ cautions (where applicable)
  - Missed dose
  - Storage

## INJECTABLE HYPOGLYCEMIC AGENTS

Instruction to the trainer: Trainer will ask the participants if they know types of injectable hypoglycemic agents. The correct answer would be injectable insulin and glucagon-like peptide 1 (GLP-1) receptor agonists. Further, trainer will ask trainee if they know different types of insulin. Trainer may allow trainee to name either generic name or brand name and onset of action. Trainer will give time to the trainee to answer the question. Then, trainer will begin the lecture using powerpoint and real objects of insulin.

### Insulin devices

Instruction to the trainer: Trainer will explore if trainees know about insulin administration technique by asking few questions to the entire group. The questions may include angle of needle during administration of insulin needle, storage of insulin among others.

### Equipment used in Diabetes Care

Instruction to the trainee: Trainer will now move on to another titled “Equipment used diabetes care”. For this, trainer will brainstorm to the trainee if they can name different equipment used in diabetes care. Trainer will give time to the trainee and will write the name of the equipment mentioned by trainee on the white board. Trainer will begin the lecture using powerpoint and different available gadgets.

## PRACTICAL WORKS

Instruction to the trainer: Trainer will arrange all the required materials to demonstrate insulin administration. The required materials are:

- - - Insulin vial
    - Insulin syringe
    - Insulin cartridge
    - Insulin pen
    - Cotton
    - Spirit
    - Sand bag
    - Maniquin

Instruction to the trainer: Trainer will let trainees re-demonstrate insulin therapy on the sand bag or maniquin

# DAY 2

## PREVIOUS DAY REVIEW

Time allocation: 30 minutes

Instruction to the trainer: Trainer will begin the day by reviewing the previous day‟s lesson briefly on risk factors, symptoms and investigations of diabetes mellitus from the previous modules. Similarly, trainer will review blood pressure on diabetes from module 2 and review on medicines from module 3. Trainer will take a short test on the previous day‟s lesson. Trainer will ask all trainee to stand up and stretch their limbs and make a sound of drizzling rain by tapping their feet on the floor for a while. Then the trainer will disclose the topic for next session as “Physical activity/ Exercise and Diabetes”.

## MODULE 4: EXERCISE REGIME FOR DM

Time allocation: 1 hour

Instruction to the trainer: Trainer will ask trainee if they do exercise and if yes what kind of exercise trainee performs. It will be voluntary for trainee to give the answer. The opportunity will be given only to two trainees. Then the trainer will begin the course content on physical activity using flex charts and powerpoint using both lecture and discussion method.

**Note for trainer:**

**Exercise Physiology** Insulin helps uptake of blood glucose by glycolysis and glycogen synthesis. Exercise promote glucose uptake both acutely and overtime by distinct mechanisms: **Contraction mediated pathway** (membrane permeability to glucose increases in glucose transporter), **Insulin stimulating pathway** (long term exercise training increases insulin sensitivity).

### Game

Time allocation: 30 mins

Instruction to the trainer: Trainer will divide trainee among three groups. Trainer will ask each group to plan and conduct physical activity that represents WHO recommendation. 5 minutes will be given to plan their physical activity. Each group will conduct one game. Each group will involve all the trainees. There will be three games in total.

**SHORT BREAK:** 5 mins

## TEA BREAK:

Time allocation: 10 minutes

## MODULE 5. DEPRESSION, STRESS AND TIME MANAGEMENT

Time allocation: 1 hours

Instruction to the trainer: Trainer will ask the trainees to share their experience related to stress which causes the depression. That may include causes of depression and stress on diabetes and how to deal with these. Trainees will learn the ways of reducing stress by streamlining their physical activity, making time to relax, getting plenty of sleep, and having a good laugh. Also trainees will examine how they are spending their time each day and brainstorm ways to improve their time-management.

## MODULE 6: DIETARY HABITS

Time allocation: 1 hours

Instruction to the trainer: Trainer will introduce the topic of diet for DM by asking trainee to share the diet pattern of the family member having diagnosed with diabetes. Trainer will give the opportunity for two trainees. Maximum 5 minutes will be allocated for one trainee. Trainer will appreciate trainee for sharing their family experience among the trainees. Then, trainer will begin the course content of the diet for the DM. Trainer will use both the lecture method and discussion method. Trainer will use charts, powerpoint, artificial diabetic plate model, real diabetes plate model, pictures of different categories of food (Importance of Whole cereals, fruits and vegetables..). Trainer will brainstorm on different categories of foods (Carbohydrate, Protein and Minerals).

Instruction to the trainer: After completing lecturer on diet, trainer will now organize a kind of game. The game needs pictures of different food items like food consisting carbohydrate, protein, vitamins, among others. Trainer will ask each trainee to select two or three pictures of food items to make a balanced diet. 30 minutes will be allocated for the whole game. Trainee will need to present their selection of food and give rationale for selecting them. Trainer will give feedback to each of the trainee based on their selection of food.

### Assignment

Instruction to the trainer: Trainer will give class assignment to the trainee. Trainer will keep the question of assignment on the board. The question will be “Plan a proper meal (4 times day) for diabetes patients)”.

## HEALTHY LUNCH BREAK

Time allocation: 45 minutes

## MODULE 7: BEHAVIOR CHANGE: ALCOHOL CONSUMPTION, SMOKING

### Alcohol and tobacco Consumption

Time allocation: 50 minutes

Instruction of the trainee: Trainers will welcome all the trainees. Trainer will allow trainees to do some warm up exercises to prevent sleepiness after lunch. After warm up exercise, trainer will now move on to another topic “ Alcohol and tobacco consumption”. Trainer will ask trainees about triggers for tobacco and alcohol consumption. Trainer will let the trainees answer on a voluntary basis. If trainees answer, then the trainer will write answers on the white board. Trainer will use a lecture method using power-point to explain how to react differently to these triggers and the health consequences of alcohol and tobacco consumption and ways to reduce intake by substituting for healthier alternatives.

### Practical work

Time allocation:1 hours

Instruction to the trainer:. Trainer will show a video on the respective education session for remaining practical work.

### Day review:

Time allocation: 10 minutes

Instruction to the trainer: Trainer will appoint two participants to wrap up the day by reviewing the module 4 and 5 and ask another participant to review the module 6 and 7. Trainer will thank all trainees for their patience to make the learning environment harmonious.

# DAY 3

### Previous day review:

Time allocation: 30 minutes

Instruction to the trainer:Trainer will greet all the trainees for a fruitful day ahead. Trainer will do a review of the previous day and take a short test of the previous lesson.

## MODULE 8: FOOT CARE

Time allocation: 1 hours

Instruction to the trainer: Trainer will ask trainees if they have seen any cases of diabetes foot in their life. If trainees say yes, the trainer will allow trainee to share his/her experience. 5 minutes will be given for sharing the experience. Later, trainer will ask trainees if they know how many diabetes patients undergo amputation in global context per minute. Trainer will appreciate trainees if they answer two diabetes patients undergo amputation every minute in the world. Trainer will further elaborate that in every 30 minutes, one diabetes patient undergoes amputation due to diabetes foot problem. Then, trainer will begin the topic properly.

Instruction to the trainer: Trainer will ask if trainees know anything about diabetes foot examination. The participation will be voluntary. Any trainee can answer if she knows the answer. Then, trainer will begin the lecture based on powerpoint.

Diabetes foot care Instruction to the trainer: Trainer will brainstorm trainees to answer one point about diabetes foot care. The answers will be written down on the board. Trainer will begin lecture based on a powerpoint and photo gallery.

### Practical work

Time allocation: 20 mins

Practical work on foot examination:

Instruction to the trainer: Trainer will arrange all the required materials for foot examination. The required materials are: Chair/ Bed Monofilament Toothpick

Instruction to the trainer: Trainer will also arrange all the required materials for foot care. The required materials are: Big bowl Luke warm water Soap Scrubber Towel Mirror Nail cutter Moisturizer

Instruction to the trainer: Trainer will ask one participant to volunteer for foot examination and care. Then trainer will ask other participants to observe demonstrations of foot examination and care. Trainer will greet the volunteer and thank her for participation. Then, trainer will explain the purpose of foot examination and care and get consent to perform the procedure. After obtaining

consent, trainer will ask the volunteer to relax herself and be seated or lie down on the bed after taking off shoe and shocks for foot examination and be seated on the chair for foot care afterwards. Trainer will perform demonstration following all the steps of foot examination and care based on the protocol attached. After demonstration, trainer will ask all the participants to do redemonstration in their respective partners.

## MODULE 9: ORAL HEALTH

Time allocation: 30 minutes

Instruction to the trainer: Trainer will begin new Module on Oral health. Trainer will ask if trainees know anything about diabetes oral examination. The participation will be voluntary. Any trainee can answer if he/she knows the answer. Then, trainer will begin the lecture based on powerpoint.

### Learning objectives of the module:

To explain the link between T2DM and Oral Health.

To elaborate most common oral health problems affecting people with diabetes. To explain important tips to help prevent oral health problems.

**SHORT BREAK:** 5 mins

## TEA BREAK:

Time allocation: 10 minutes

## MODULE 10: SEXUAL HEALTH

Time allocation: 30 minutes

Instruction to the trainer: Trainer will move on to another session on sexual health. Trainer will ask if trainees know anything about diabetes sexual health. The participation will be voluntary. Any trainee can answer if he/she knows the answer. Then, trainer will begin the lecture based on powerpoint.

### Learning objectives of the module:

To describe the Sexual health in DM

To explain how to deal with sexual problem

## MODULE 11: TRAVEL TIPS AND SICK DAYS

Instruction to the trainer: Trainer will begin Module on Travel tips and sick days. Trainer will ask if trainees know how to take care of diabetes during travel and sick days. The participation will be voluntary. Any trainee can answer if he/she knows the answer. Then, the trainer will begin the lecture based on powerpoint.

## TRAVEL TIPS:

Time allocation: 30 minutes

### Learning objectives of the module:

To elaborate how to stay well during travel and during flight

## SICK DAYS:

Time allocation: 20 minutes

### Learning objectives of the module:

To explain what to do when unwell

To clarify when to call a doctor if unwell.

## MODULE 12: SOCIAL AND EMOTIONAL SUPPORTS / MEET TO THE HEALTH PROFESSIONAL

**SOCIAL AND EMOTIONAL SUPPORTS:**

Time allocation: 30 minutes

Instruction to the trainer: Trainer will now move to the last module on social and emotional supports. Trainer will ask trainees if they know the importance of social and emotional support for diabetes. Any trainee can answer if he/she knows the answer. Then, trainer will begin the lecture based on powerpoint.

### Learning objectives of the module:

To explain the need of social and emotional support to diabetes To explore the tips to care and support to diabetic patient

## HEALTHY LUNCH BREAK

Time allocation: 45 minutes

## MEET TO THE HEALTH PROFESSIONAL:

Time allocation: 20 minutes

Instruction to the trainer: Trainer will continue module on meet to the health professional. Trainer will ask if trainees know when to meet and how often diabetic patient should meet health professional. The participation will be voluntary. Any trainee can answer if he/she knows the answer. Trainer will appreciate trainees if they answer. Then, trainer will begin the lecture based on powerpoint.

### Learning objectives of the module:

To explain the need of a visit to a health professional by a diabetes patient.

### Practical work

Time allocation: 40 mins

Instruction to the trainer:. Trainer will show a video on the respective education session for remaining practical work.

### Post Test

Time allocation: 20 minutes

## CLOSING CEREMONY

Instruction to the trainer: Trainer will prepare for closing ceremony before hand. Preparation should include printing of certificates, allocating Master of ceremony for the closing ceremony, invitation to the guests for the closing ceremony; arrange stage for the program among others. At the end of the ceremony, a group photo will be taken.

Instruction to the trainer: Trainer will review the whole training session. Trainer will collect feedback from each trainee in a paper. Feedback should include both positive feedback and feedback for areas to be improved for upcoming training.

**Schedule of closing ceremony**

| Activities | Remarks |
| --- | --- |
| Welcoming all the participants |  |
| Offering seats |  |
| Welcome speech |  |
| Remarks from the chief guest |  |
| Remarks from the organizer |  |
| Remarks from representative of the trainers |  |

| Experience sharing from representative of the trainees |  |
| --- | --- |
| Certificate distribution |  |
| Closing remarks |  |
| Group photo |  |

## REFERENCES

1. Renwick C., Riddell M., Engel L., Reddy P., Oldenburg B., and the Australasian Peers for Progress Diabetes Project (2010). “The Australasian Peers for Progress Diabetes Project Participant Workbook. Monash University: Melbourne; ISBN:978-09804308-8-7
2. Fayfman M, Pasquel FJ, Umpierrez GE. Management of Hyperglycemic Crises. Medical Clinics. 2017;101(3):587-606.
3. Stratton IM, Adler AI, Neil HAW, Matthews DR, Manley SE, Cull CA, et al. Association of glycaemia with macrovascular and microvascular complications of type 2 diabetes (UKPDS 35): prospective observational study. Bmj. 2000;321(7258):405-12.
4. Kitabchi AE, Umpierrez GE, Murphy MB, Barrett EJ, Kreisberg RA, Malone JI, et al. Management of hyperglycemic crises in patients with diabetes. Diabetes care. 2001;24(1):131- 53.
5. Umpierrez G, Korytkowski M. Diabetic emergencies—ketoacidosis, hyperglycaemic hyperosmolar state and hypoglycaemia. Nature reviews Endocrinology. 2016;12(4):222.
6. Dhatariya KK, Vellanki P. Treatment of diabetic ketoacidosis (DKA)/hyperglycemic hyperosmolar state (HHS): novel advances in the management of hyperglycemic crises (UK versus USA). Current diabetes reports. 2017;17(5):33. 6. Association AD.
7. Glycemic Targets: Standards of Medical Care in Diabetes—2018. Diabetes care. 2018;41(Supplement 1):S55-S64.
8. Association AD. 10. Microvascular Complications and Foot Care: Standards of Medical Care in Diabetes—2018. Diabetes care. 2018;41(Supplement 1):S105-S18.
9. Boulton AJ, Armstrong DG, Albert SF, Frykberg RG, Hellman R, Kirkman MS, et al. Comprehensive foot examination and risk assessment: a report of the task force of the foot care

interest group of the American Diabetes Association, with endorsement by the American Association of Clinical Endocrinologists. Diabetes care. 2008;31(8):1679-85.

1. Monteiro‐ Soares M, Dinis‐ Ribeiro M. A new diabetic foot risk assessment tool: DIAFORA. Diabetes/metabolism research and reviews. 2016;32(4):429-35.
2. Laakso M. Cardiovascular disease in type 2 diabetes: challenge for treatment and prevention. Journal of internal medicine. 2001;249(3):225-35.
3. Juutilainen A, Lehto S, Rönnemaa T, Pyörälä K, Laakso M. Type 2 diabetes as a ―coronary heart disease equivalent‖: an 18-year prospective population-based study in Finnish subjects. Diabetes care. 2005;28(12):2901-7.
4. Association AD. 9. Cardiovascular Disease and Risk Management: Standards of Medical Care in Diabetes—2018. Diabetes care. 2018;41(Supplement 1):S86-S104.
5. Association AD. 8. Pharmacologic Approaches to Glycemic Treatment: Standards of Medical Care in Diabetes—2018. Diabetes care. 2018;41(Supplement 1):S73-S85.
6. Thiruvoipati T, Kielhorn CE, Armstrong EJ. Peripheral artery disease in patients with diabetes: Epidemiology, mechanisms, and outcomes. World journal of diabetes. 2015;6(7):961.
7. Marso SP, Hiatt WR. Peripheral arterial disease in patients with diabetes. Journal of the American College of Cardiology. 2006;47(5):921-9.
8. Association AD. Peripheral arterial disease in people with diabetes. Diabetes care. 2003;26(12):3333-41.
9. Zhou H, Zhang X, Lu J. Progress on diabetic cerebrovascular diseases. Bosnian journal of basic medical sciences. 2014;14(4):185.
10. Ergul A, Kelly-Cobbs A, Abdalla M, C Fagan S. Cerebrovascular complications of diabetes: focus on stroke. Endocrine, Metabolic & Immune Disorders-Drug Targets 2012;12(2):148-58.
11. Bruno A, Liebeskind D, Hao Q, Raychev R, Investigators US. Diabetes mellitus, acute hyperglycemia, and ischemic stroke. Current treatment options in neurology. 2010;12(6):492-503.
12. Chen R, Ovbiagele B, Feng W. Diabetes and stroke: epidemiology, pathophysiology, pharmaceuticals and outcomes. The American journal of the medical sciences. 2016;351(4):380- 6
13. WHO. Oral Health. Available at:<http://www.euro.who.int/en/health-> topics/diseaseprevention/oral-health
14. <https://www.ncbde.org/certification_info/what-is-a-cde/>

**Other references:** <https://medlineplus.gov/ency/patientinstructions/000082.htm> <https://medlineplus.gov/ency/patientinstructions/000082.htm> <https://medlineplus.gov/ency/patientinstructions/000082.htm>

The Nutritional Source [Internet]. 2016 [cited 2016, August]. Available from: <https://www.hsph.harvard.edu/nutritionsource/healthy-eating-plate-vs-usda-myplate>

1. DIABETES AUSTRALIA

Diabetes Australia is the voice for people living with diabetes, their families and carers. [www.diabetesaustralia.com.au](http://www.diabetesaustralia.com.au/)

Tel: (03) 9667 1777

Infoline: 1300 136 588
